# A MALAT1/HIF-2α feedback loop contributes to arsenite carcinogenesis

**DOI:** 10.18632/oncotarget.6806

**Published:** 2015-12-31

**Authors:** Fei Luo, Baofei Sun, Huiqiao Li, Yuan Xu, Yi Liu, Xinlu Liu, Lu Lu, Jun Li, Qingling Wang, Shaofeng Wei, Le Shi, Xiaolin Lu, Qizhan Liu, Aihua Zhang

**Affiliations:** ^1^ Institute of Toxicology, School of Public Health, Nanjing Medical University, Nanjing 211166, Jiangsu, People's Republic of China; ^2^ The Key Laboratory of Modern Toxicology, Ministry of Education, School of Public Health, Nanjing Medical University, Nanjing 211166, Jiangsu, People's Republic of China; ^3^ The Key Laboratory of Environmental Pollution Monitoring and Disease Control, Ministry of Education, School of Public Health, Guiyang Medical University, Guiyang 550025, Guizhou, People's Republic of China; ^4^ Department of Epidemiology and Biostatistics, School of Public Health, Nanjing Medical University, Nanjing 211166, Jiangsu, People's Republic of China; ^5^ Thoracic and GI Oncology Branch, Center for Cancer Research, National Cancer Institute, Bethesda, MD 20892, USA

**Keywords:** lncRNAs, HIFs, arsenite, carcinogenesis

## Abstract

Arsenic is well established as a human carcinogen, but the molecular mechanisms leading to arsenic-induced carcinogenesis are complex and elusive. It is also not known if lncRNAs are involved in arsenic-induced liver carcinogenesis. We have found that MALAT1, a non-coding RNA, is over-expressed in the sera of people exposed to arsenite and in hepatocellular carcinomas (HCCs), and MALAT1 has a close relation with the clinicopathological characteristics of HCC. In addition, hypoxia-inducible factor (HIF)-2α is up-regulated in HCCs, and MALAT1 and HIF-2α have a positive correlation in HCC tissues. During the malignant transformation of human hepatic epithelial (L-02) cells induced by a low concentration (2.0 μM) of arsenite, MALAT1 and HIF-2α are increased. In addition, arsenite-induced MALAT1 causes disassociation of the von Hippel-Lindau (VHL) protein from HIF-2α, therefore, alleviating VHL-mediated HIF-2α ubiquitination, which causes HIF-2α accumulation. In turn, HIF-2α transcriptionally regulates MALAT1, thus forming a positive feedback loop to ensure expression of arsenite-induced MALAT1 and HIF-2α, which are involved in malignant transformation. Moreover, MALAT1 and HIF-2α promote the invasive and metastatic capacities of arsenite-induced transformed L-02 cells and in HCC-LM3 cells. The capacities of MALAT1 and HIF-2α to promote tumor growth are validated in mouse xenograft models. In mice, arsenite induces an inflammatory response, and MALAT1 and HIF-2α are over-expressed. Together, these findings suggest that the MALAT1/HIF-2α feedback loop is involved in regulation of arsenite-induced malignant transformation. Our results not only confirm a novel mechanism involving reciprocal regulation between MALAT1 and HIF-2α, but also expand the understanding of the carcinogenic potential of arsenite.

## INTRODUCTION

Arsenite, an environmental compound with distinct physical characteristics and toxicity, is a human carcinogen that is recognized for its importance in public health [1]. Epidemiological evidence shows that chronic exposure to inorganic arsenic induces cancers, including lung, liver, and bladder cancers. It also induces neoplastic transformation of human cells, and such models have been used to investigate the mechanisms of arsenite-induced carcinogenesis. [2–4]. Transcription factors and miRNAs are involved in arsenite-induced neoplastic transformation of cells [5], but their mechanisms remain largely uninvestigated.

Hypoxia inducible factors (HIFs), HIF-α and HIF-β, control the expression of hundreds of genes that function in oncogenic pathways and in the regulation of proliferation, apoptosis, and tumor metabolism [6]. To date, investigations have largely focused on the regulation of protein-coding genes for these pathways [7]. However, new sequencing technologies are identifying non-coding transcripts with regulatory roles that are also relevant to cancer biology [8, 9]. Many of these non-coding genes are also regulated by hypoxia, and, in particular, long non-coding RNAs (lncRNAs) are regulated by HIF transcriptional pathways.

lncRNAs are a new class of RNAs, with lengths ranging from 200 bp to 100 kbp. Dysregulation of lncRNAs has been implicated in a variety of human diseases, including cancer [10]. lncRNAs contribute to transcriptional regulation by modulating the activity of transcription factors and by serving as scaffolds for assembling transcriptional regulators [11]. Post-transcriptional functions for lncRNAs are now known, with examples of lncRNAs titrating miRNAs and RNA-binding proteins, modulating mRNA decay and suppressing target mRNA translation, and functioning as a platform for protein ubiquitination [12–14].

Three lncRNAs, named HOXA transcript at the distal tip (HOTTIP) [15], highly upregulated in liver cancer (HULC), and high expressed in HCC (HEIH), have functions in hepatocellular carcinomas (HCCs) [16]. Opposite to these tumor promotion functions of lncRNAs, the lncRNA metallothionein 1D pseudogene (MT1DP) acts as a tumor suppressor, for its over-expression results in reduced cell proliferation and colony formation in soft agar and increased apoptosis in liver cancer cells [17].

Despite studies demonstrating that expression profiles for HIFs and lncRNAs correlate with tumor growth, limited information is available regarding mechanisms by which alterations in lncRNAs and HIFs contribute to initiation and early progression of arsenite-induced malignancies. In the present study, L-02 cells in culture and in animal model systems were utilized to examine lncRNA alterations mediated by arsenite. Chronic exposure of L-02 cells to arsenite induced malignant transformation and induced lncRNAs, which enhanced accumulation of HIFs. In turn, HIFs regulated transcription of lncRNAs, providing evidence for the existence of a feedback loop between HIFs and lncRNA that promotes arsenite-induced malignant transformation of L-02 cells. Further, arsenite induced over-expression of HIF-2α and MALAT1 in animals and enhanced the inflammatory response. The results provide insight into the mechanisms of how lncRNAs/HIFs contribute to arsenite-induced liver carcinogenesis.

## RESULTS

### Arsenite exposure is associated with liver and kidney damage

Blood samples (nonpatient, *n* = 16; and patient, *n* = 16) were examined to measure the extent of exposure and to assess liver and kidney damage in those exposed to arsenite (Table 1). Relative to the control group, urinary and hair arsenite concentrations were higher (*p* < 0.01, Table 1). Consistent with the difference of arsenite exposure, the albumin/globulin (A/G) ratio, an indicator of liver damage, was lower in the exposed group relative to the control group (*p* < 0.01; Table 1). In addition, the BUN levels, which indicate kidney damage, of the exposed group were higher than those for the control group (*p* < 0.05; Table 1). These results indicate that arsenite exposure is associated with liver and kidney damage.

**Table 1:** Liver and kidney damage (mean ± SD) in villagers from Guizhou Province (control and exposed groups)

Group	As (mean ± SD)		Liver damage			Kidney damage	
	Urine (μg/L)	Hair (μg/g)	A/G	ALT (U/L)	AST (U/L)	BUN (mmol/L)	CREA (μmol/L)
Control	20.8 ± 7.8	0.1 ± 0.07	1.6 ± 0.19	20.9 ± 4.9	28.3 ± 5.19	4.5 ± 0.82	68.0 ± 11.10
Exposed	45.4 ± 19.4**	0.4 ± 0.2**	1.3 ± 0.23**	25.1 ± 11.80	35.3 ± 15.43	5.7 ± 1.18*	63.4 ± 12.76

Abbreviations: As, arsenic; A/G, the ratio of albumin and globulin; ALT, glutamic-pyruvic transaminase; AST, glutamic-oxalacetic transaminase; BUN, blood urea nitrogen; CREA, creatinine.

** *P* < 0.01, significantly different compared with the control group.

* *P* < 0.05, significantly different compared with the control group

ICP-MS was used to measure both urinary arsenic and hair arsenic levels in all subjects.

### lncRNAs are over-expressed in sera of patients exposed to arsenite

**Figure 1:**
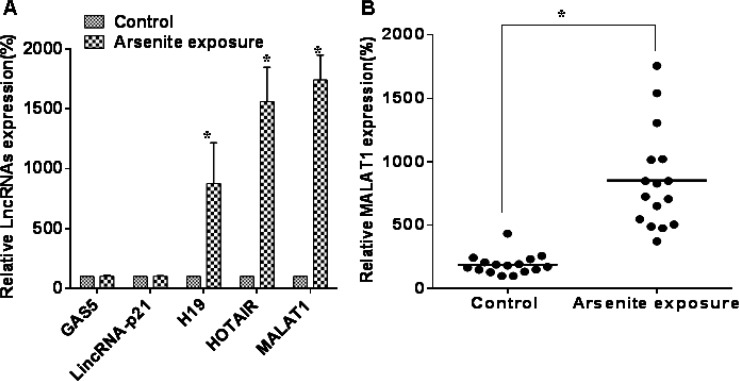
Some lncRNAs are over-expressed in sera of people exposed to arsenite (**A**) Serum levels of lncRNAs, GAS5, lincRNA-p21, H19, HOTAIR, and MALAT1 were determined by qRT-PCR assays (means ± SD, *n* = 3) in those exposed to arsenite (*n* = 16) or not exposed (*n* = 16). *P < 0.05 different from control. (**B**) The levels of MALAT1 were determined by qRT-PCR assays (means ± SD, *n* = 3) in those exposed to arsenite (*n* = 16) or not exposed (*n* = 16). *P < 0.05 different from control.

The expression of lncRNAs in sera of those exposed and not exposed to arsenite was measured. To assess candidate lncRNAs for functional studies, we determined if some common lncRNAs were differentially expressed in the sera of those exposed to arsenite. H19, HOTAIR, and MALAT1 were higher in the sera of 16 persons with long-term exposure to arsenite than in the sera of 16 controls; of the three lncRNAs, the differential expression of MALAT1 was highest (Figure 1A and 1B). These results show that some lncRNAs are over-expressed in sera of people with long-term exposure to arsenite.

### In HCC specimens, the levels of MALAT1 are high, and patients with lower levels of MALAT1 have longer survival times

**Figure 2:**
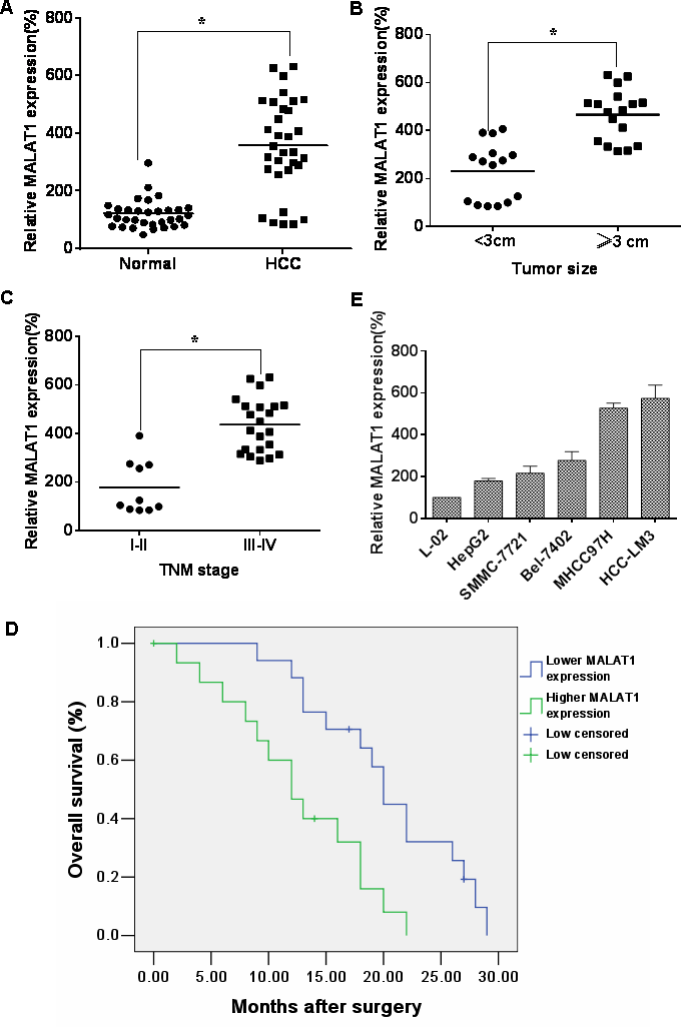
MALAT1 over-expression is associated with clinicopathological characteristics of HCC (**A**) The levels of MALAT1 in HCC tissues (*n* = 32) and in paired adjacent normal tissues (*n* = 32) were determined by qRT-PCR assays (means ± SD, *n* = 3) *P < 0.05 different from adjacent normal tissues. (**B**) The levels of MALAT1 were determined by qRT-PCR assays (means ± SD, *n* = 3) in tumors < 3 cm (*n* = 15) and in tumors ≥ 3 cm (*n* = 17). *P < 0.05, different from tumors < 3 cm. (**C**) The mRNA levels of MALAT1 (means ± SD, *n* = 3) in tumors of patients with clinical stage I–II (*n* = 10) and with clinical stage III–IV (*n* = 22) were measured by qRT-PCR assays. *P < 0.05, different from clinical stages I–II. Kaplan-Meier curves were constructed for survival of patients with HCC cancers divided according to the levels of MALAT1. (**D**) There were significantly (*P* = 0.005) shorter survival times for patients with high MALAT1 levels than for those with low MALAT1 levels. (**E**) The MALAT1 levels were determined by qRT-PCR (means ± SD, *n* = 3) for HCC cell lines (HepG2, SMMC-7721, Bel-7402, MHCC97H, and HCC-LM3) with different aggressive characteristics. L-02 cells were used as control cells.

The expression of MALAT1 is up-regulated in cancers of the lung, breast, pancreas, liver, colon, uterus, cervix and prostate [18]. To determine if MALAT1 is differentially expressed in HCC tissues, 32 paired HCC tissues and adjacent normal tissues were analyzed for the levels of MALAT1. In HCC specimens, relative to adjacent normal liver tissues, MALAT1 levels were up-regulated (Figure 2A). As with most solid tumors, there is a hypoxic microenvironment in HCCs [19], and HIFs are involved in the pathogenesis and pathophysiology of HCCs [20]. As determined in the present experiments, HIF-2α was over-expressed in 32 paired HCC tissues compared to adjacent normal liver tissues (Supplementary Figure S1A and S1B), and there was a positive correlation between MALAT1 and HIF-2α in HCC tissues (Supplementary Figure S1C). In addition, the correlations of MALAT1 expression with clinicopathological parameters (i.e., maximum diameter, TNM stage) were used to assess their clinical significance. Tumors > 3 cm had high MALAT1 expression (Figure 2B), and the levels of MALAT1 were higher with increasing clinical stage (Figure 2C). The clinicopathological characteristics of the patients are listed in Table 2. The levels of MALAT1 in HCCs were not associated with other parameters, such as age (*p* = 0.500) or gender (*p* = 0.576) (Table 2). These results indicate that, in HCC specimens, the levels of MALAT1 are over-expressed and that they correlate with the clinicopathological characteristics of HCC.

**Table 2:** Correlation between the levels of MALAT1 and the clinicopathological characteristics of HCC

Characteristic	MALAT1		
	High (*N* = 15)	Low (*N* = 17)	*P*
**Age (years)**			
≦ 50	7	9	0.500
> 50	8	8	
**Gender**			
Male	12	13	0.576
Female	3	4	
**HBsAg^a^**			
Positive	13	14	0.563
Negative	2	3	
**Serum AFP^b^ (ng/ml) < 200**	1	5	0.116
> 200 11 15	11	15	
**Cirrhosis**			
Yes	13	12	0.254
No	2	5	
**Tumor size**			
< 3 cm	3	12	0.005*
≥ 3 cm	12	5	
**Multinodular tumor**			
Yes	12	9	0.108
No	3	8	
**TNM stage**			
I–II	1	9	0.006*
III–IV	14	8	

a HBsAg: surface antigen of the hepatitis B virus.

b AFP: α-fetoprotein, a marker for liver cancer.

* *P* < 0.05.

To determine the relationship between MALAT1 levels and the prognosis for HCC patients, the correlation between MALAT1 expression and overall survival (OS) was evaluated by Kaplan–Meier analysis. The OS at 5 years for patients with low MALAT1 expression was higher than that for those with high MALAT1 expression (Figure 2D). The longer survival for HCC patients with lower levels of MALAT1 indicates that MALAT1 is a prognostic indicator for OS of patients with HCC. The expression of MALAT1 in five cell lines derived from liver cancer cells was also examined. MALAT1 levels in the normal liver L-02 cells were lower than those in HepG2 and Bel-7402 (weakly malignant), SMMC-7721 (moderately malignant), MHCC97H and HCC-LM3 (highly malignant) cells. Moreover, there were higher levels of MALAT1 in more aggressive cells; the highest levels were in the HCC-LM3 cells (Figure 2E). These data show that the levels of MALAT1 are differentially expressed in relation to the aggressive characteristics of liver cancer.

### In L-02 cells, arsenite-induced neoplastic transformation has effects on the levels of lncRNAs and HIF-2α

To investigate the effects of arsenite on cell proliferation and cell transformation, L-02 cells were exposed to 1.0, 2.0, 5.0, 10.0, or 20.0 μM arsenite for 24, 48, or 72 h. Relative cell proliferation was increased in cells incubated with 1.0 and 2.0 μM arsenite at 24 h but was decreased by 10.0 and 20.0 μM arsenite at 48 h and 72 h. There were no appreciable effects of 5.0 μM arsenite on cell proliferation (Supplementary Figure S2A). Relative to the control, there was an initial increase in the growth of cells incubated with 2.0 μM arsenite. Therefore, we chose 2 μM arsenite for use in following experiments. L-02 cells were exposed to 0.0 or 2.0 μM arsenite for about 15 weeks (30 passages). The numbers of transformed cells increased relative to control cells (Supplementary Figure S2B).

**Figure 3:**
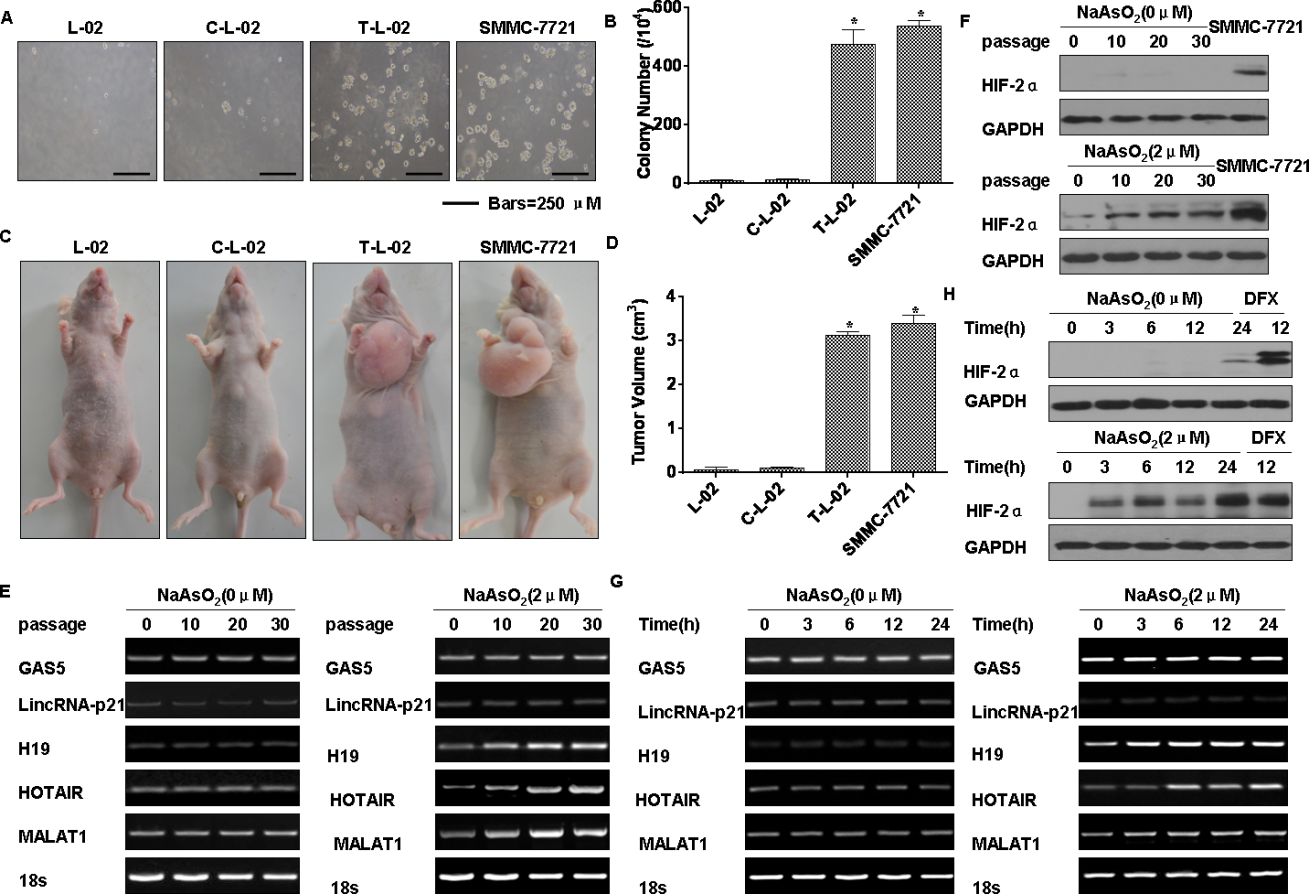
Arsenite-induced neoplastic transformation has effects on the levels of lncRNAs and HIF-2α in L-02 cells *Abbreviations*: *L-02*, normal L-02 cells, *C-L-02*, passage control L-02 cells; *T*-*L-02*, arsenite-transformed L-02 cells; *SMMC-7721*, SMMC-7721 carcinoma cells. Densities of bands were quantified by Eagle Eye II software. GAPDH levels, measured in parallel, served as controls. L-02 cells were exposed to 0.0 or 2.0 μM of arsenite for 30 passages. (**A**) Colonies of normal L-02 cells, passage control L-02 cells, arsenite-transformed L-02 cells, and SMMC-7721 cells and their numbers (**B**) (means ± SD, *n* = 3) in soft agar (bars *=* 250 μm). *P < 0.05 different from medium control cells. (**C**) At 4 weeks after inoculation of normal L-02 cells, passage control L-02 cells, arsenite-transformed L-02 cells, and SMMC-7721 cells, tumors that formed from the transformed cells and SMMC-7721 cells were examined, and their volumes (**D**) were measured (means ± SD, *n* = 6). *P < 0.05 different from medium control cells. L-02 cells were exposed to 0.0 or 2.0 μM of arsenite for 0, 10, 20, or 30 passages. (**E**) RT-PCR analyses of the mRNA levels of GAS5, lincRNA-p21, H19, HOTAIR, and MALAT1. (**F**) Western blots for HIF-2α were made for L-02 cells exposed to 0.0 or 2.0 μM arsenite for 0, 3, 6, 12, or 24 h. (**G**) RT-PCR was performed for GAS5, lincRNA-p21, H19, HOTAIR, and MALAT1, and (**H**) Western blots for HIF-2α were made.

To determine if cells chronically exposed to arsenite had acquired anchorage-independent growth capacity, their capacity for such growth was evaluated. In agar, colonies were formed by L-02 cells exposed to 2.0 μM arsenite, and colonies were formed by the carcinogenic SMMC-7721 cells. In contrast, control cells showed no anchorage-independent growth (Figure 3A and 3B). In addition, tumor incidences in the groups of mice injected with arsenite-transformed L-02 cells and SMMC-7721 carcinoma cells were 100% (6/6 per group); for the control group, the incidence was 0% (0/6). The tumor volumes for the groups implanted with arsenite-transformed cells and SMMC-7721 cells were larger. (Figure 3C and 3D).

The expressions of various lncRNAs in L-02 cells exposed to 0.0 or 2.0 μM arsenite for 0, 10, 20, or 30 passages were assessed. MALAT1, H19, and HOTAIR were increased in arsenite-transformed L-02 cells, and their expressions increased with increased numbers of passages (Figure 3E). Similar to our previous results showing that expression of HIF-2α is induced in human bronchial epithelial cells exposed to arsenite [4], we demonstrated that HIF-2α is up-regulated in HCC tissues. We also determined that HIF-2α expression in L-02 cells increases with increased numbers of passages in the presence of arsenite (Figure 3F). The expressions of MALAT1, H19, and HOTAIR increased after arsenite exposure over periods ranging from 0 to 24 h (Figure 3G). Over 24 h, the levels of HIF-2α were also increased by arsenite (Figure 3H). Furthermore, HIF-2α was expressed in L-02 cells exposed for 12 h to DFX, an iron chelator used to mimic hypoxia [21] (Figure 3H). Thus, a low level of arsenite induces malignant transformation of L-02 cells and up-regulates MALAT1 and HIF-2α.

### Effect of MALAT1 on the degradation of HIF-2α in L-02 cells exposed to arsenite

**Figure 4:**
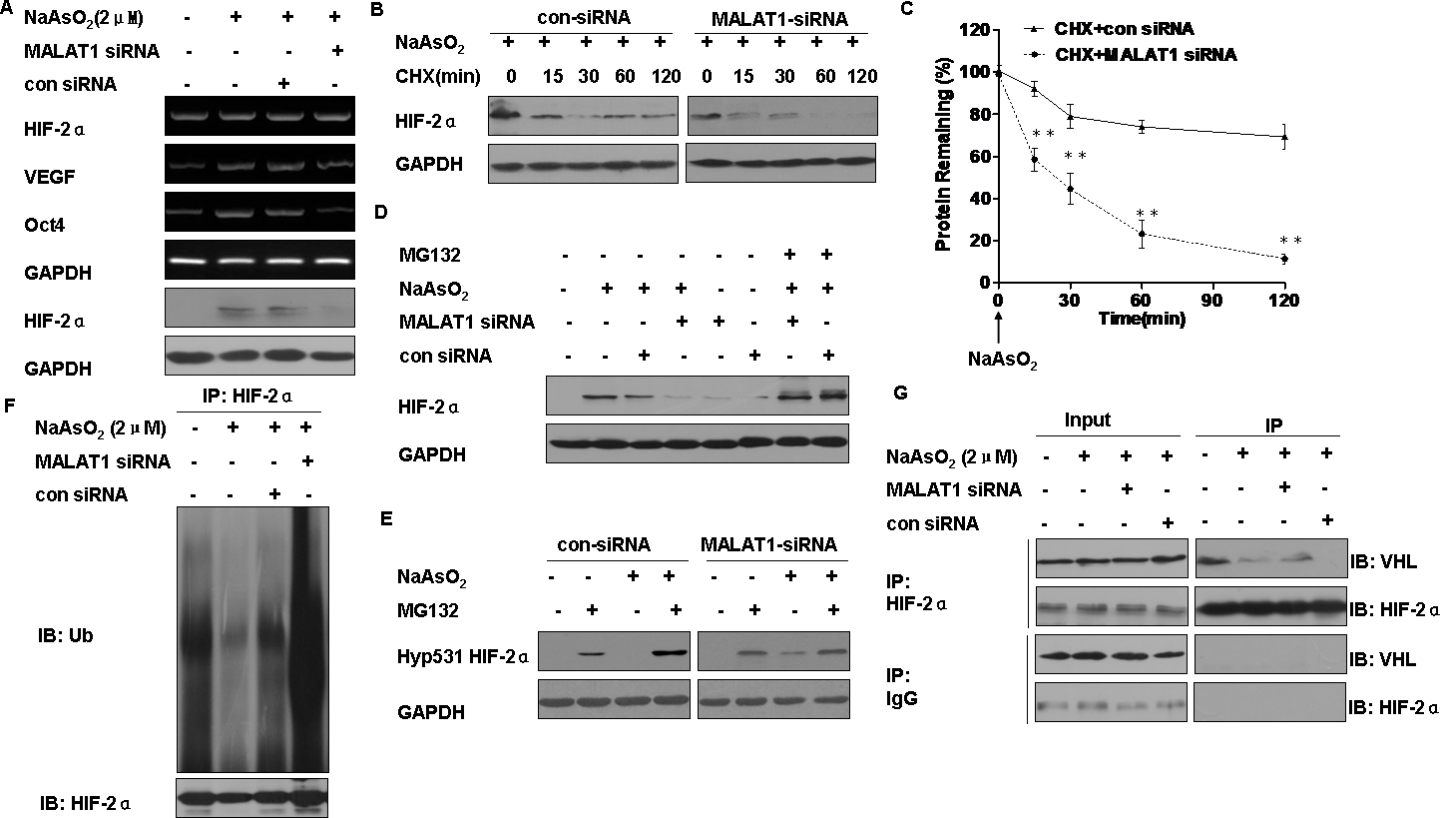
Functions of MALAT1 in the degradation of HIF-2α in L-02 cells exposed to arsenite L-02 cells were exposed to 100 ppm of control siRNA or MALAT1 siRNA for 24 h, then incubated with 0.0 or 2.0 μM arsenite for 24 h. (**A**) The mRNA levels of HIF-2α, Oct-4, and VEGF were measured by RT-PCR, and Western blots of HIF-2α were made. L-02 cells were exposed to 100 ppm of control siRNA or MALAT1 siRNA for 24 h, then exposed to 2.0 μM arsenite for 24 h before they were treated with the protein synthesis inhibitor, CHX (20 μg/ml) for the indicated periods of time. (**B**) Western blots were made, and (**C**) protein expression (means ± SD, *n* = 3) of HIF-2α was determined. **P < 0.01 different from cells treated with control siRNA. L-02 cells were treated with control siRNA or MALAT1 siRNA for 24 h then exposed to 0.0 or 10.0 μM proteasome inhibitor MG132 in the absence or presence of 2.0 μM arsenite for 24 h. (**D**) The levels of HIF-2α were analyzed by Western blots. (**E**) The levels of Hyp531 HIF-2α were analyzed by Western blots. L-02 cells were exposed to 100 ppm of control siRNA or MALAT1 siRNA for 24 h, then incubated with 0.0 or 2.0 μM arsenite for 24 h. (**F**) The levels of HIF-2α were determined by Western blots after total protein of cells was subjected to co-immunoprecipitation with HIF-2α (IP) and ubiquitin (IB) antibodies. (**G**) Western blot analyses of HIF-2α and VHL after cell lysates were subjected to co-immunoprecipitation with HIF-2α (IP) and VHL (IB) antibodies.

We next determined if MALAT1 mediates HIF-2α expression. After transfection of L-02 cells exposed to arsenite for 24 h with MALAT1-specific siRNA1, siRNA2, or siRNA3, levels of MALAT1 mRNA decreased (Supplementary Figures S3A and S3B). Of these, siRNA3 was more efficient, and it was applied in further experiments. In L-02 cells exposed to arsenite, HIF-2α mRNA levels were not affected, however, there was a decrease of HIF-2α protein expression and of HIF-2α gene targets; mRNA levels of *VEGF* and *Oct4* were decreased by knockdown of MALAT1 (Figure 4A). These results indicate that HIF-2α accumulation mediated by MALAT1 upon arsenite exposure occurs at the posttranscriptional level. Then, we found that arsenite could stable HIF-2α protein in the cellular (Supplementary Figure S3C and S3D). Next, in the arsenite-treated cells transfected with control siRNA, the levels of HIF-2α protein decreased slowly. In contrast, in the MALAT1-transfected cells, the HIF-2α protein had a shorter half-life and was barely detectable at 30 min (Figure 4B and 4C).

To determine if the faster reduction of HIF-2α protein in MALAT1-inhibited cells was associated with its proteasome-dependent degradation. The reduction of HIF-2α caused by MALAT1 knockdown was reversed by MG132 (Figure 4D). Since hydroxylation of the HIF-2α protein at proline residue 531 is required for VHL binding and subsequent degradation through the ubiquitin-proteasome pathway [22], the effect of MALAT1 on hyp531 HIF-2α levels was determined. MALAT1 knockdown led to an increase in hyp531 HIF-2α levels in L-02 cells exposed to arsenite (Figure 4E). Further, knockdown of MALAT1 attenuated arsenite-reduced HIF-2α polyubiqutination (Figure 4F). Moreover, knockdown of MALAT1 enhanced the HIF-2α-VHL interaction in L-02 cells exposed to arsenite (Figure 4G). Together, these results indicate that arsenite-induced MALAT1 causes disassociation of VHL from HIF-2α, thereby alleviating VHL-mediated HIF-2α ubiquitination and subsequent degradation.

### MALAT1 is a transcriptional target of HIF-2α in L-02 cells exposed to arsenite

**Figure 5:**
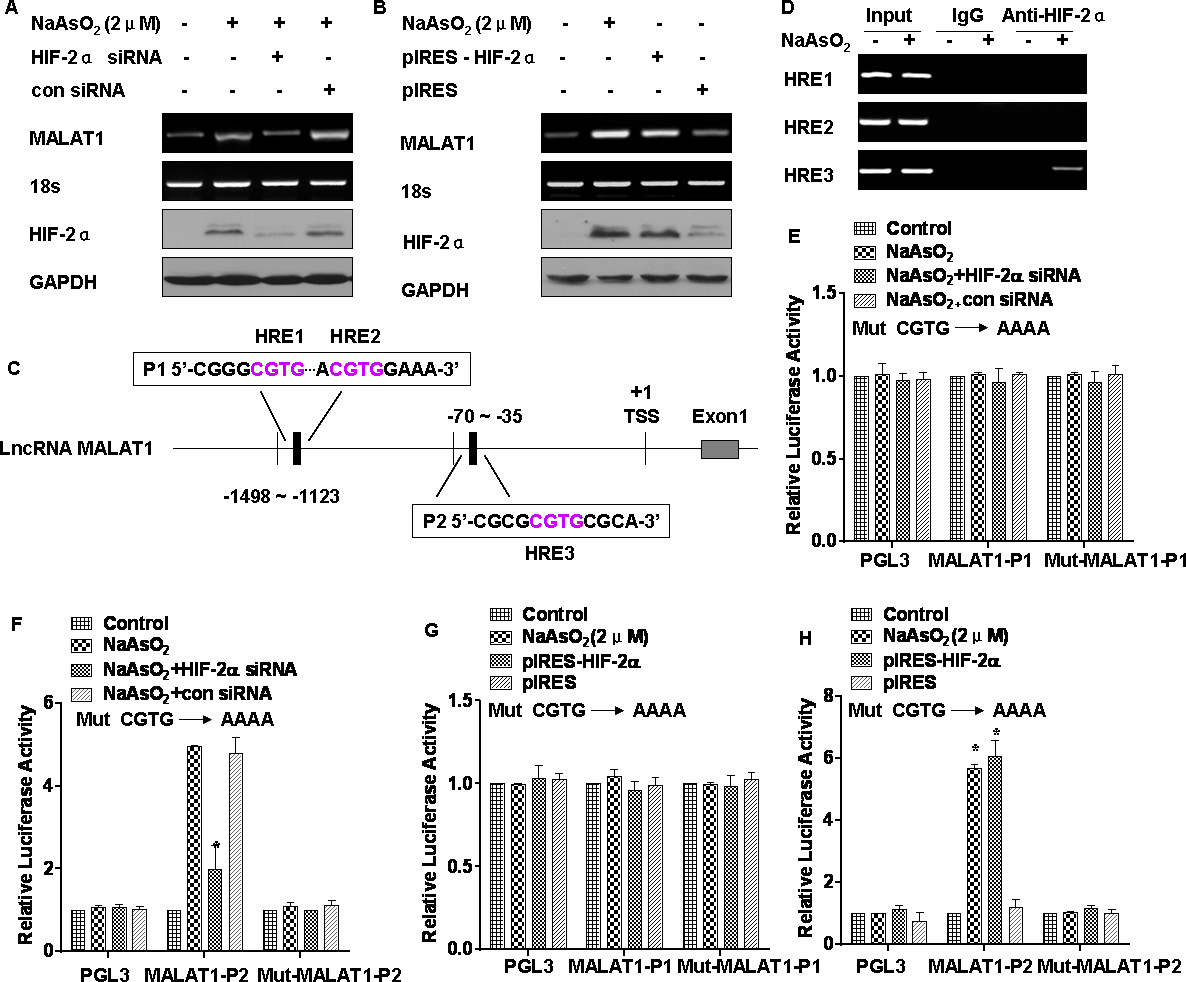
MALAT1 is regulated by HIF-2α in L-02 cells exposed to arsenite GAPDH or 18 s ribosomal RNA, measured in parallel, served as controls. L-02 cells were exposed to 20 nM of control siRNA or to 10 nM HIF-2α siRNA for 24 h, and then incubated with 0.0 or 2.0 μM arsenite for 24 h. (**A**) The mRNA levels of MALAT1 were determined by RT-PCR, and the levels of HIF-2α were analyzed by Western blot analyses. L-02 cells were transfected with the HIF-2α plasmid for 24 h, then incubated with 0.0 or 1.0 μM arsenite for 24 h. (**B**) The mRNA levels of MALAT1 were determined by RT-PCR, and the levels of HIF-2α were analyzed by Western blot analyses. (**C**) Schematic illustration of the consensus HIF-2α HREs in the *MALAT1* gene promoter. L-02 cells were treated with 0.0 or 2.0 μM arsenite for 24 h. (**D**) The binding of HIF-2α to promoters of MALAT1, three domains of HRE, was measured by a ChIP assay after the chromatin was immunoprecipitated with antibodies against HIF-2α. L-02 cells were co-transfected with HIF-2α siRNA and reporter constructs for 24 h, then incubated with 0.0 or 1.0 μM arsenite for 24 h. (**E**) Luciferase activities of MALAT1-P1 and Mut-MALAT1-P1, and (**F**) MALAT1-P2 and Mut-MALAT1-P2 were measured and normalized to Renilla luciferase activity (means ± SD, *n* = 3); *P < 0.05 different from control cells treated with arsenite. L-02 cells were co-transfected with the HIF-2α plasmid and reporter constructs for 24 h, then incubated with 0.0 or 1.0 μM arsenite for 24 h. (**G**) Luciferase activities of MALAT1-P1 and Mut-MALAT1-P1, and (**H**) MALAT1-P2 and Mut-MALAT1-P2 were measured and normalized to Renilla luciferase activity (means ± SD, *n* = 3); *P < 0.05 different from control cells.

In endothelial cells, hypoxia increases MALAT1, which controls a phenotypic switch [23]. We hypothesized that MALAT1 is regulated by HIF-2α. To establish this, HIF-2α was knocked down in L-02 cells exposed to arsenite. In these cells, knockdown of HIF-2α attenuated arsenite-induced MALAT1 up-regulation (Figure 5A). Conversely, ectopic expression of HIF-2α increased MALAT1 expression equivalent to arsenite-induced MALAT1 upregulation (Figure 5B). These data indicate that HIF-2α is responsible for arsenite-induced MALAT1 expression.

We next determined if HIF-2α regulates MALAT1 expression at the transcriptional level. The genomic sequence upstream of the gene coding for MALAT1 was inspected by use of the Genomatix suite of sequence analysis tools (MatInspector). Three putative hypoxia response elements (HREs, 5′-RCGTG-3′) were found within the promoter of the *MALAT1* gene (Figure 5C). Chromatin immunoprecipitation (ChIP) assays were used to determine the association of HIF-2α and the chromatin fragments corresponding to the three HREs within the MALAT1 gene. For L-02 cells exposed to arsenite, the antibody against HIF-2α immunoprecipitated DNA fragments containing the potential binding site, which was the third HRE, not the first and the second HREs in the promoter regions of MALAT1 (Figure 5D). We also confirmed an interaction of HIF-2α with the third HRE of MALAT1 promoter regions in HCC-LM3 cells (Supplementary Figure S4A). In addition, we evaluated whether the HREs within the MALAT1 gene confer HIF-2α-dependent transcriptional activity. Since the first and second HREs were close, one reporter plasmid containing the two HREs was constructed and designated as MALAT1-P1; the other one was designated as MALAT1-P2. DNA fragments containing wild-type or mutant HREs were inserted into the promoter region of a luciferase reporter plasmid. The result was that luciferase expression from MALAT1-P2, but not from the MALAT1-P1 reporter or the mutant reporter, was decreased by inhibiting expression of HIF-2α (Figure 5E and 5F). In contrast, luciferase expression from MALAT1-P2, but not the MALAT1-P1 reporter or the mutant reporter, was induced by arsenite and by ectopic expression of HIF-2α (Figure 5G and 5H). These results demonstrate that MALAT1 is transcriptionally upregulated by HIF-2α in L-02 cells exposed to arsenite.

### MALAT1 and HIF-2α are involved in the arsenite-induced malignant transformation of L-02 cells

**Figure 6:**
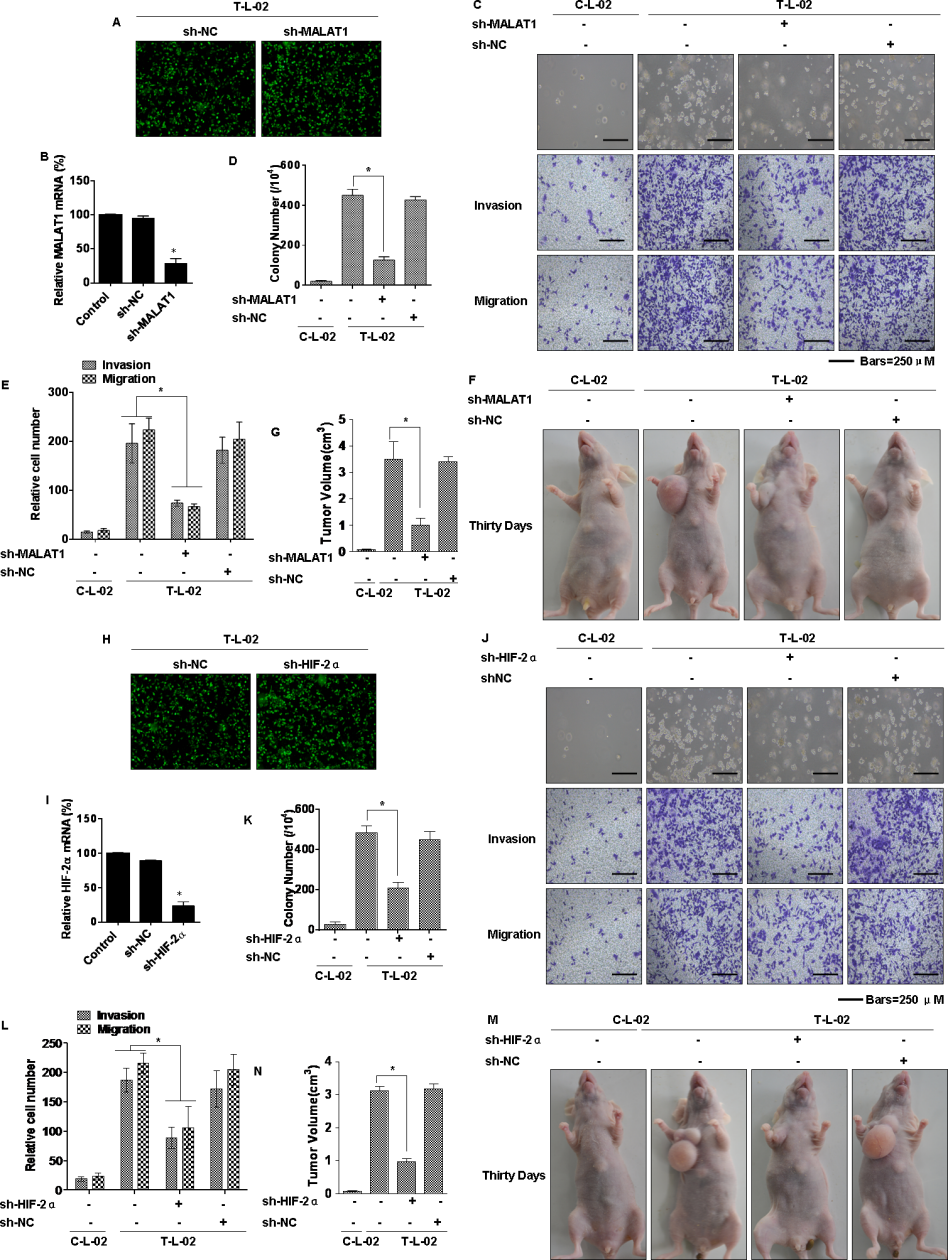
Influence of MALAT1 and HIF-2α on the neoplastic capacity of transformed L-02 cells *Abbreviations*: *C-L-02*, passage control L-02 cells; *T*-*L-02*, arsenite-transformed L-02 cells. T-L-02 cells were infected with a non-targeting control vector (sh-NC) or MALAT1 shRNA (sh-MALAT1), inducing puromycin resistance. Cells were cultured for at least 2 weeks in the presence of puromycin (5 μg/mL) before the following experiments. (**A**) T-L-02/shNC cells and T-L-02/sh-MALAT1 cells. Fluorescent microscopy. Sh-MALAT1 and sh-NC were transfected into T-L-02 cells. At 24 h after transfection, fluorescent microscopy showed emission green fluorescence. (**B**) qRT-PCR validated the downregulation of MALAT1 after shRNA knockdown in T-L-02 cells, *P < 0.05 different from T-L-02 cells. (**C**) Colony formation was assessed in soft agar (above), and representative images of cells migration and cell invasion (middle and bottom) and (**D**) their colony numbers and (**E**) relative migrating/invading cells (means ± SD, *n* = 3) were quantified, bars = 250 μm. *P < 0.05 different from T-L-02 cells. 1 × 10^7^ cells were injected into nude mice (*n* = 6). (**F**) Tumor volumes were determined (**G**) at 4 weeks after injection, (*n* = 6, mean ± SD), *P < 0.05 different from T-L-02 cells. T-L-02 cells were infected with a non-targeting control vector (sh-NC) or HIF-2α shRNA (sh-HIF-2α), inducing puromycin resistance. Cells were cultured for at least 2 weeks in the presence of puromycin (5 μg/mL) before the following experiments. (**H**) T-L-02/sh-NC cells and T-L-02/sh-HIF-2α cells. Fluorescent microscopy. Sh-HIF-2α and sh-NC were transfected into T-L-02 cells. At 24 h after transfection, fluorescent microscopy showed emission green fluorescence. (**I**) qRT-PCR validated the downregulation of HIF-2α after shRNA knockdown in T-L-02 cells, *P < 0.05 different from T-L-02 cells. (**J**) Colony formation was assessed in soft agar, and representative images of cell migration and cell invasion and (**K**) their colony numbers and (**L**) relative migrating/invading cells (means ± SD, *n* = 3) were quantified, bars = 250 μm. *P < 0.05 different from T-L-02 cells. 1 × 10^7^ cells were injected into nude mice (*n* = 6). (**M**) Tumor volumes were determined (**N**) at 4 weeks after injection, (*n* = 6, mean ± SD), *P < 0.05 different from T-L-02 cells.

Since MALAT1 and HIF-2α are over-expressed in arsenite-transformed L-02 cells (Figure 3), the functions of MALAT1 and HIF-2α during arsenite-induced malignant transformation, an early step in liver carcinogenesis, were examined. Arsenite-transformed L-02 cells were transduced with lentivirus-mediated sh-MALAT1 or sh-RNA negative control (shRNA-NC) vectors. The transduced cells were detected as green fluorescent protein (GFP)-positive cells following the addition of GFP together with the MALAT1 shRNAs. The expression of the lentivirus-mediated MALAT1 shRNAs was confirmed by fluorescence imaging (Figure 6A). The level of MALAT1 detected by qRT–PCR was reduced following transduction by MALAT1 shRNAs compared with that in the arsenite-transformed L-02 cells and shRNA-NC-transduced cells (Figure 6B). These findings confirmed that the lentivirus-mediated transduction of the MALAT1 shRNAs were effective. Incubated under anchorage-independent conditions, the T-L02-sh-NC cells produced an elevated number of large colonies, but cells silenced for MALAT1 formed only a few colonies, and these were of small size (Figure 6C, upper, and 6D). To determine if MALAT1 facilitates migration and invasion of arsenite-transformed L-02 cells, invasion through Matrigel and migration through Transwells were evaluated. Relative to controls, inhibition of MALAT1 impeded the invasion and migration of arsenite-transformed L-02 cells (Figure 6C, below, and 6E). These effects of MALAT1 on malignant and metastatic capacity were confirmed in HCC-LM3 cells, which were also transfected with lentivirus sh-MALAT1 (Supplementary Figures S5A, S5B, S5C, S5D, and S5E). Moreover, MALAT1-knockdown cells were injected into nude mice. MALAT1 knockdown decreased the sizes of tumors that developed relative to the arsenite-treated group (Figure 6F and 6G). MALAT1 knockdown, however, did not change the incidence rate. In addition, the lentivirus-negative vector (sh-NC) and sh-HIF-2α were transfected into arsenite-transformed L-02 cells. At post-transfection, fluorescent microscopy showed emission of green fluorescence (Figure 6H). qRT-PCR showed that expression of HIF-2α level was decreased in sh-HIF-2α-transfected, arsenite-transformed L-02 cells (T-L-02-sh-HIF-2α cells), compared to control cells (Figure 6I). Silencing of HIF-2α reduced the formation of colonies and inhibited the invasion and migration in arsenite-transformed cells (Figure 6J, 6K and 6L). The role of HIF-2α in the malignant and metastatic capacity was confirmed in HCC-LM3 cells, which were also transfected with sh-HIF-2α (Supplementary Figures S5F, S5G, S5H, S5I and S5J). HIF-2α-knockdown cells were injected into nude mice, HIF-2α knockdown decreased the tumor sizes relative to the arsenite-treated group (Figure 6M and 6N). These data indicate that MALAT1 and HIF-2α are involved in the neoplastic and metastatic capacity of arsenite-transformed L-02 cells.

### Arsenite-induced inflammatory response and effects on the levels of HIF-2α and MALAT1 in mice

**Figure 7:**
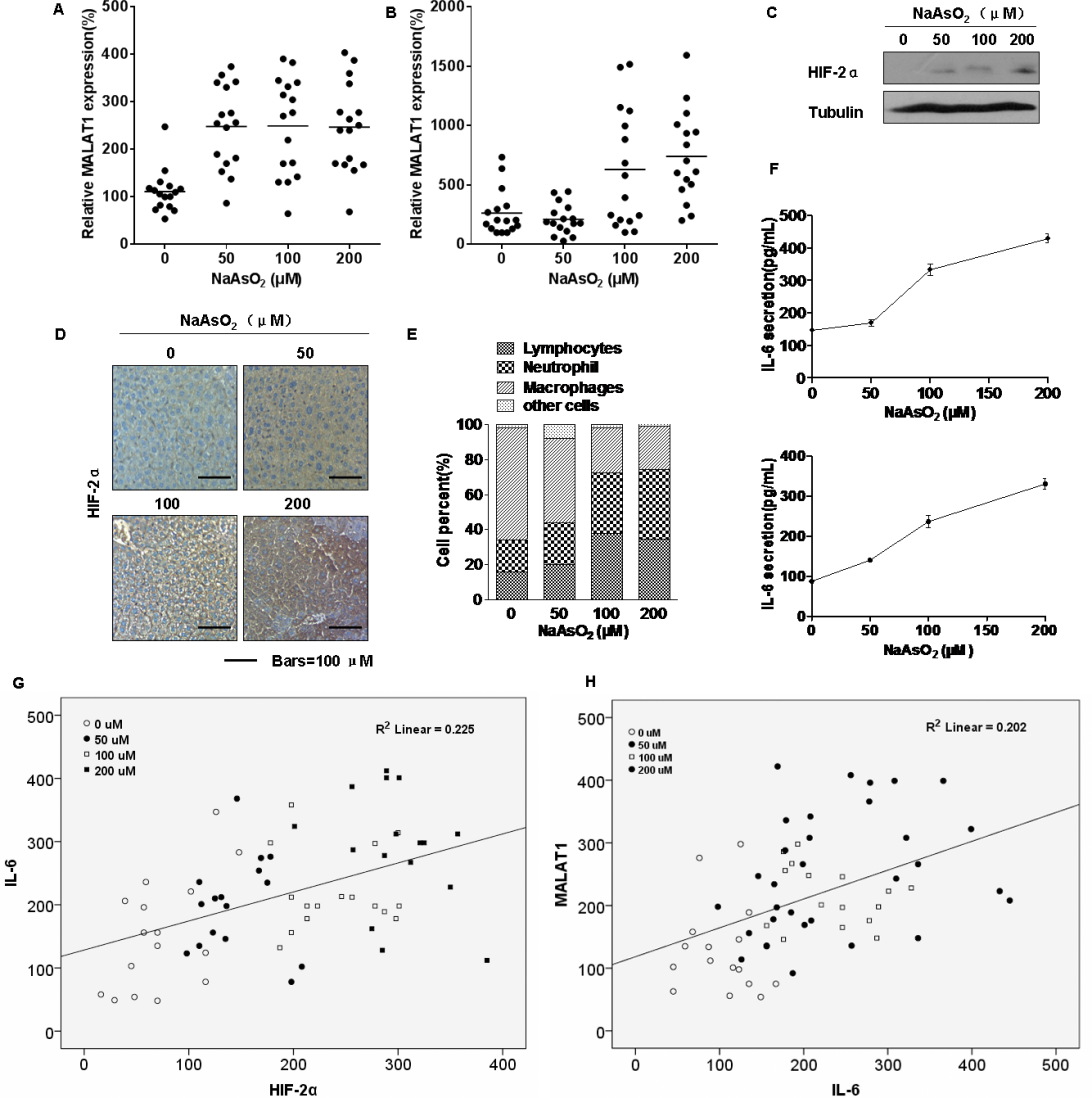
Effect of arsenite on the levels of HIF-2α and MALAT1 in mice GAPDH levels, measured in parallel, served as controls. The four groups of CD1 mice were untreated or treated with low, middle, or high concentrations (50 μM, 100 μM, or 200 μM, respectively) of arsenite, which were added to the drinking water daily for 3 months. (**A** and **B**) By qRT-PCR assays, the levels of MALAT1 were determined (means ± SD, *n* = 3) in sera of animals dosed with arsenite (*n* = 16) and in hepatic tissues of different groups of animals. (**C**) Western blots for HIF-2α were made for hepatic tissues of the different groups of animals. (**D**) Immunohistochemical analyses of HIF-2α levels in the hepatic tissues of mice treated with low, middle, or high concentrations (50 μM, 100 μM, or 200 μM, respectively) of arsenite. (**E**) The BALF cellularity patterns of the groups of CD1 mice untreated or treated with arsenite. (**F**) The levels of IL-6 present in the mouse mice BALF (top) and serum (bottom) (means ± SD, *n* = 3) were measured by ELISA. (**G**) The relationship between IL-6 levels and HIF-2α levels in the tissues of mice untreated or treated with low, middle, or high concentrations (50 μM, 100 μM, and 200 μM, respectively) of arsenite (*n* = 16, *R* ^2^ = 0.225). (**K**) The relationship between MALAT1 levels and IL-6 levels in the tissues of mice untreated or treated with low, middle, or high concentrations (50 μM, 100 μM, 200 μM, respectively) of arsenite (*n* = 16, *R*^2^ = 0.202).

The expressions of MALAT1 and HIF-2α were evaluated in mice. Four groups of CD1 mice were untreated or treated with low, middle, or high concentrations (50 μM, 100 μM, and 200 μM, respectively) of arsenite, which were added to the drinking water daily for three months. There were no appreciable differences in body weights of the four groups (Supplementary Table 2). However, the expression of MALAT1 in sera and liver tissues of mice exposed to arsenite was increased (Figure 7A and 7B, respectively), as were HIF-2α levels in liver tissues of these mice (Figure 7C and 7D). Our previous study showed that chronic exposure of cultured HBE cells to arsenite induces an inflammatory response associated with HIF-2α, which contributes to arsenite-induced malignant transformation [24]. Although we failed to detect tumors in mice exposed to arsenite, we found that the numbers of macrophages, lymphocytes, and neutrophils in the broncho-alveolar lavage fluid (BALF) of the arsenite-treated group were higher than those for the control group (Figure 7E and Table 3). Further, release of the pro-inflammatory IL-6 cytokine into the BALF and serum increased (Figure 7F, top and bottom, respectively). The mRNA levels and release of other pro-inflammatory cytokines (TNF-α and IL-8) from liver tissue, serum, and BALF increased, but there was no change for IL-1β (Supplementary Figures S6A, S6B, S6C and S6D). These results indicate that arsenite induces inflammation in mice. The expression of IL-6 positively correlated with the levels of HIF-2α in liver tissues (Figure 7G). Further, there was a positive correlation between MALAT1 and IL-6 levels (Figure 7H). Thus, arsenite induces over-expression of HIF-2α and MALAT1 in mice and may cause an elevated inflammatory response.

**Table 3:** Effect of arsenite on the recruitment of inflammatory cells into BALF (total inflammatory cells and absolute counts/ml)

Dose (μM)	Total cells (× 10^3^/ml)	Macrophages (× 10^3^/ml)	Lymphocytes (× 10^3^/ml)	Neutrophils (× 10^3^/ml)
0	25.9 ± 2.51	16.5 ± 0.21	4.1 ± 1.1	4.8 ± 1.7
50	40.1 ± 1.52**	19.2 ± 2.14**	8.1 ± 1.77**	9.5 ± 1.4**
100	72.33 ± 3.7**	18.5 ± 3.43**	27.3 ± 4.76**	25.8 ± 5.1**
200	81.2 ± 4.73**	19.8 ± 2.41**	28.1 ± 3.1**	32.3 ± 4.3**

** *P* < 0.01, different from control group.

## DISCUSSION

Inorganic arsenite is a widely distributed, naturally occurring environmental contaminant affecting tens of millions of people worldwide [25]. Chronic exposure to arsenite causes carcinogenesis of lung, skin, liver, and bladder [26]. Various molecular mechanisms have been proposed for arsenite-induced carcinogenesis [27], but the function of lncRNAs in arsenite-induced cancer has not previously been evaluated.

As early as 1976, villagers from Guizhou province in southwestern China were reported to be suffering severe symptoms of arsenicosis, which was attributed to exposure to high levels of arsenic in food, especially in corn and chili peppers, and to a lesser extent by breathing arsenic-laden air [28]. miRNAs can be used as biomarkers of disease as well as for arsenic exposure, and can account for disease etiology [29]. Nevertheless, whether or not lncRNAs are involved in arsenite carcinogenesis has remained unknown. The levels of the lncRNA HOTAIR are increased in primary breast tumors and metastases, and its level in primary tumors is a predictor of metastasis and death [15]. MALAT1 is an lncRNA associated with metastasis and survival in early-stage non-small cell lung tumors (NSCLCs) [30]. In some prostate and breast cancer cell lines, expression of GAS5 induces growth arrest and apoptosis independent of other stimuli [31]. After genotoxic stress, lincRNA-p21 is up-regulated in breast cancer cells [32], indicating that it is associated with survival of these cells. Based on their functions, lncRNAs can be classified into oncogene and tumor-suppressor types [33]. In the present study, we collected 32 villagers sera in December 2013, and determined the expression of some common lncRNAs. In those arsenite exposure samples, the arsenite concentrations in urine and hair were higher than those not exposed, and those who were exposed had kidney and liver damage. We also found that, of lncRNAs with elevated expression, MALAT1 had the highest expression. These data indicate that lncRNAs may serve as serum biomarker for diagnosing exposure to arsenite.

lncRNAs are defined as non-coding RNA transcripts longer than 200 nt. Thus far, more than 10, 000 lncRNAs have been identified in the human genome [34]. MALAT1 is an lncRNA originally found to be over-expressed in patients at high risk for metastasis of NSCLCs [30]. MALAT1 is up-regulated in human lung cancers, breast cancers, pancreatic cancers, colon cancers, prostate cancers, and endometrial stromal sarcomas [30, 35]. For several cancers, MALAT1 expression is an independent prognostic parameter for survival [18]. Initially, we determined MALAT1 expression in HCC cell lines, HCC tissues, and in paired adjacent normal tissues and found that its expression was higher in HCC cell lines and HCC tissues. We also found that HIF-2α was over-expressed in 32 paired HCC tissues relative to adjacent normal liver tissues. For HCC patients, high MALAT1 expression was associated with greater tumor size, higher stage, and shorter OS. These results suggest that MALAT1 functions as an oncogene in liver cancer.

Chronic exposure to low levels of arsenite (< 5 μM) causes cell proliferation, which can lead to neoplastic transformation, whereas high levels cause cytotoxicity, indicating that the effects of arsenite are dependent on the degree of exposure [3, 36]. In the United States and China, the current maximum contaminant level for arsenite in drinking water is 10 μg/L (∼ 0.5 μM) [37]. In this study, 1.0 and 2.0 μM concentrations of arsenite, lower than levels in drinking water in areas where arsenicosis is common, did not cause cell death [38]. Since the cell proliferation rate induced by 2.0 μM arsenite was higher than that induced by 1.0 μM arsenite, we used 2.0 μM arsenite for long-term exposures. This low level of arsenite enhanced neoplastic transformation of cells, as determined by anchorage-independent growth in soft agar and tumorigenesis in nude mice. In addition, with increased time of exposure to arsenite, there were more malignant cells and elevated expression of MALAT1. Based on these data, we suggest that MALAT1 may be involved in the neoplastic transformation of L-02 cells induced by arsenite.

Under normoxia, the HIF-α protein is maintained at low levels due to continuous synthesis and degradation, but levels increase rapidly in response to hypoxia [39]. HIF-α is not only induced by hypoxia, but is activated in normoxic cells in response to various stresses [40]. For example, exposure of normoxic cells to peptide mediators, including insulin and IL-1, stabilizes the HIF-1α protein [41], which is involved in nickel-induced malignant transformation of cells under normoxic conditions [42]. Under normoxic conditions, arsenite and some other metals directly (catalytically) or indirectly induce the stabilization and transactivation of HIF-1α [5]. The present studies show elevated expression of HIF-2α in normoxic cells with increased time of exposure to arsenite. Thus, these data indicate that HIF-2α may be involved in the neoplastic transformation of L-02 cells induced by arsenite.

Although mammalian lncRNAs are best known for modulating transcription, they have a post-transcriptional influence on mRNA splicing, stability, and translation [43]. An example is that HOTAIR facilitates the ubiquitination of ataxin-1 by Dzip3 and snurportin-1 by mex3b in cells and accelerates their degradation [14]. Moreover, lincRNA-p21 causes disassociation of VHL from HIF-1α, thereby alleviating VHL-mediated HIF-1a ubiquitination and subsequent degradation [13]. Under hypoxic conditions, HIFs accumulate in a posttranslational manner. Under normoxic conditions, the capacity of HIFs to activate transcription is prevented through its hydroxylation. Hydroxylated HIFs recruit the VHL tumor suppressor, leading to the recognition by E3 ubiquitin ligase and subsequent ubiquitination and proteasome-dependent degradation [44]. The arsenite-induced accumulation of HIF apparently relates to posttranscriptional regulation [5].

Since, in the present research, both MALAT1 and HIF-2α were induced by arsenite in L-02 cells, we speculated that MALAT1 regulates HIF-2α degradation through the ubiquitin-proteasome pathway. Consistent with this hypothesis, HIF-2α mRNA levels were not affected by knockdown of MALAT1, but there was a decrease of HIF-2α protein in L-02 cells exposed to arsenite, moreover, mRNA levels of HIF-2α target genes, *VEGF* and *Oct4*, were decreased. The HIF-2α reduction caused by MALAT1 knockdown was rescued by the proteasome inhibitor MG132. Moreover, reduced degradation of hyp531 HIF-2α enhanced the HIF-2α-VHL interaction and increased HIF-2α polyubiqutination. Thus, arsenite-induced MALAT1 causes disassociation of VHL from HIF-2α and thereby alleviates VHL-mediated HIF-2α ubiquitination and subsequent degradation.

Similar to human lincRNA-p21, which is induced by hypoxia, other lncRNAs, including H19 and lncRNA-low expression in tumor (LET), are regulated by hypoxia and are involved in hypoxia-induced signaling transduction in cancer [45, 46]. These observations, together with ours, suggest that lncRNAs are involved in the regulation of hypoxia-induced signaling induced by arsenite. HIFs transcriptionally regulate expression of a variety of genes by binding to HREs in their promoters [47]. In the present study, analysis of the gene promoter region (MatInspector) allowed us to predict that there are three putative HREs (5′-*RCGTG*-3′) in gene promoter region of the *MALAT1* gene, suggesting that HIF-2α regulates MALAT1 expression via binding to HREs. We determined the association of HIF-2α and the chromatin fragments corresponding to the third HRE within the MALAT1 gene in L-02 cells exposed to arsenite and in HCC-LM3 cells. Further, inhibition of HIF-2α decreased expression of MALAT1, and HIF-2α upregulated MALAT1-HRE3 transcriptional activity. These results indicate that MALAT1 is transcriptionally up-regulated by HIF-2α in L-02 cells exposed to arsenite.

MALAT1 was originally identified as an lncRNA, showing high expression in individuals at high risk for metastasis of non-small cell lung tumors [6]. Its expression is upregulated in a range of tumors, including lung cancer, liver cancer, renal cell carcinoma, bladder cancer, and osteosarcoma [48]. HIF-2α is involved in tumor cell proliferation, apoptosis, migration, and invasion and in the metastatic spread of tumor cells [47]. Our studies focused on the effects of MALAT1 and HIF-2α on the neoplastic and invasive capacity of cells after long-term exposure to arsenite. Knockdown of MALAT1 with lentivirus shRNA inhibited the neoplastic and invasive capacity of arsenite-transformed L-02 cells and HCC-LM3 cells. The roles of HIF-2α in solid tumors have been assessed [47]. We also found that knockdown of HIF-2α with lentivirus shRNA inhibited the neoplastic and invasive capacity of arsenite-transformed L-02 cells and HCC-LM3 cells. These results indicate that MALAT1 and HIF-2α are involved in the neoplastic and metastatic capacity of arsenite-transformed L-02 cells and HCC-LM3 cells.

Finally, we determined that MALAT1 and HIF-2α were up-regulated in mice exposed to arsenite for three months. MALAT1 was over-expressed in mouse serum and liver tissues, and HIF-2α levels were elevated in liver tissues of these mice. Low-dose arsenic exposures (5–250 ppb in drinking water, or nM to low μM concentrations) increased neovascularization of chicken chorioallantoic membranes, stimulated inflammatory angiogenesis in a mouse Matrigel assay, and increased vascular density and vessel size in mouse tumors [49]. Our previous study showed that chronic arsenite exposure of cultured HBE cells induces an inflammatory response, which contributes to arsenite-induced malignant transformation [24]. We have shown that arsenite induces inflammation in mice, but does not cause tumors. Michael P. Waalkes research team confirmed that lung, liver, gallbladder, and ovarian tumors could be induced by “whole-life” inorganic arsenic exposure in CD1 mice at human-relevant doses [50, 51]. Although short time exposure to arsenic did not induce tumors, our results could provide a hint that MALAT1 and HIF-2α over-expression were involved in arsenite carcinogenesis. In the future, we plan to implement whole-life exposure of mice to arsenite and determine the relationship between arsenite carcinogenesis and HIF-2α and MALAT1. In addition, the expression of IL-6 positively correlated with the levels of HIF-2α in liver tissues of mice. Further, there was a positive correlation between MALAT1 and IL-6 in liver tissues of mice. These data indicate that arsenite induces HIF-2α and MALAT1 over-expression in animals and induces an inflammatory response.

In summary, we have shown that MALAT1, a non-coding RNA, was over-expressed in the sera of people exposed to arsenite and in hepatocellular carcinomas (HCCs), and MALAT1 had a close relation with the clinicopathological characteristics of HCC. In addition, hypoxia-inducible factor (HIF)-2α was up-regulated in HCCs, and MALAT1 and HIF-2α had a positive correlation in HCC tissues. In addition, in arsenite-induced neoplastic transformation of L-02 cells, over-expression of MALAT1 increased HIF-2α expression through the ubiquitin-proteasome pathway. In turn, HIF-2α transcriptionally regulated MALAT1, thus forming a positive feedback loop to ensure arsenite-induced MALAT1 and HIF-2α expression, which were involved in malignant transformation and carcinogenesis. We also found that, in mice, arsenite induced an inflammatory response and over-expression of MALAT1 and HIF-2α. Together, these findings suggest that the MALAT1/HIF-2α feedback loop was involved in regulation of arsenite-induced malignant transformation. Our results not only confirm a novel mechanism involving reciprocal regulation of MALAT1 and HIF-2α and also expand the understanding of the carcinogenic potential of arsenite.

## MATERIALS AND METHODS

### Biological samples

We consulted a database maintained by the Guizhou Provincial Office of Endemic Disease to identify populations exposed to arsenite [52]. In December 2013, our team collected samples from the target population, with a total of 32 villagers agreeing to participate in the study. Their participation was approved by our institutional review board. Informed consent could not be offered, because the data were analyzed anonymously. Arsenicosis symptoms were categorized based on the degree of symptoms: nonpatient (*n* = 16) and severe patient (*n* = 16). Symptoms were classified according to the Chinese National Arsenicosis Diagnosis Standard protocol [52]. Patients (*n* = 16), defined as individuals showing symptoms of arsenicosis, from Jiaole, Guizhou province, were designated the arsenite exposure group. The other 16 villagers, from near Jiaole who were not exposed to arsenite and had no symptoms of arsenicosis, were as designated the control group (Table 1).

### Patients and tissue samples

A total of 32 Chinese HCC patients were involved. Consent was obtained for all patients. These patients underwent curative liver resection for primary tumors between March 2009 and July 2012 in the Department of General Surgery, The Second Affiliated Hospital, Nanjing Medical University. The inclusion criteria for the patient cohort included (i) having a distinctive pathological diagnosis of HCC; (ii) surgical resection, defined as complete resection of all tumor nodules with the cut margin being free of cancer by histological examination; and (iii) having complete clinicopathological data. An exclusion criterion was having anticancer treatment before liver resection. None of the patients had extrahepatic metastases when they underwent hepatectomy. The clinicopathological characteristics of the patients are listed in Table 2. This study was reviewed and approved by Medical Ethics Committee of the Second Affiliated Hospital of Nanjing Medical University.

### Animals

Mice (CD1) were purchased from Shanghai Laboratory Animal Center, Shanghai, China), and housed in the animal facilities at the Jiangsu Center for Disease Control and Prevention. Animals were treated humanely and with regard for alleviation of suffering according to a protocol approved by the Jiangsu Center for Disease Control and Prevention Institutional Animal Care and Use Committee. Briefly, 64 male CD1 mice aged 7–8 weeks were randomly divided into four groups (16 mice per group). One group was reserved as the non-treated control group. The other three groups were treated with low, middle, and high concentrations of arsenite (50 μM, 100 μM, and 200 μM, respectively), which was added to the drinking water daily for three months.

### Cell culture and reagents

L-02 cells, a normal human liver cell line, and HCC cell lines (HepG2, SMMC-7721, Bel-7402, MHCC97H, and HCC-LM3) were obtained from the Shanghai Institute of Cell Biology, Chinese Academy of Sciences (Shanghai, China) and were maintained in 5% CO_2_ at 37°C in RPMI-1640 or DMEM medium, respectively, supplemented with 10% fetal bovine serum (FBS, Life Technologies/Gibco, Grand Island, NY), 100 U/ml penicillin, and 100 μg/ml streptomycin (Life Technologies/Gibco, Gaithersburg, MD). For chronic exposure, 1 × 10^6^ L-02 cells were seeded into 10-cm (diameter) dishes for 24 h and maintained in 0.0 or 2.0 μM sodium arsenite (NaAsO_2_, Sigma, St. Louis, MO; purity, 99.0%) for 48–72 h per passage. This process was continued for about 15 weeks (30 passages); desferroxamine (DFX), an iron chelator routinely used to mimic hypoxia, was purchased from Sigma. The proteasome inhibitor, MG132, and the protein synthesis inhibitor, cycloheximide (CHX), were purchased from Calbiochem (Darmstadt, Germany). All other reagents were of analytical grade or the highest grade available.

### Serum RNA extraction

Blood samples were centrifuged at 15000g to separate the sera. All of the samples were collected and stored at −70^°C^ and thawed immediately before assay. Total RNA was isolated from serum using TRIzol LS reagent (Invitrogen, Life Technologies, Paisley, UK) according to manufacturer's instructions with the following modifications: In brief, each 250 μl serum sample was mixed with 750 μl TRIzol LS Reagent. After 5 min incubation at room temperature, 200 μl of chloroform was added, followed by 15 sec of shaking and 10 min of incubation at room temperature. The mixture was centrifuged at 12, 000g for 15 min at 4^°C^ in a concentrator (Eppendorf-Netheler-Hinz, Hamburg, Germany). The aqueous layer containing RNA was transferred into a new tube, then RNA was precipitated for 16 hr at −20°C with 0.5 ml isopropyl alcohol and washed with 1 ml of 75% ethanol. Finally, the RNA pellet was dried for 5–10 min at room temperature, dissolved at 60^°C^ in 15 μl of RNase-free water. The RNA concentration was measured with a NanoDrop spectrophotometer (Thermo Fisher Scientific). The final concentrations of RNA ranged from 319∼782 ng/μl. The TRIzol method is described in other studies [53, 54].

### Reverse-transcriptase polymerase chain reaction (RT-PCR)

Total cellular and tissue RNA was isolated by use of TRIzol (Invitrogen) according to the manufacturer's recommendations. Total RNA (2 μg) was transcribed into cDNA by the use of AMV reverse transcriptase (Promega, Madison, Wisconsin, USA). Primers used are listed in Supplementary Table 1. PCR was evaluated by checking the products on 2% w/v agarose gels. Bands were quantified by densitometry and normalized by the use of glyceraldehyde 3-phosphate dehydrogenase (GAPDH) or 18s ribosomal RNA to correct for differences in loading. For densitometric analyses, the mRNA bands on the gels were measured by Eagle Eye II.

### Quantitative real-time PCR (qRT-PCR)

The levels of lncRNAs were determined by qRT-PCR. 18s ribosomal RNA was used as a control. Forward (F) and reverse (R) primers were as follows: *MALAT1-F, 5′- ACTACCAGCCATTTCTCC -3′; MALAT1-R, 5′- ACC ACCACAGGTTTACAG -3′; 18s-F, 5′- GTAACCCGTTGAA CCCCATT -3′; 18s-R, 5′- CCATCCAATCGGTAGTAGCG -3′. GAS5-F, 5′-CAATAGATTCCTTCGCTCC-3′; GAS5-R, 5′-AGTTCACCTCTGGGTTTCA-3′. LincRNA-p21-F, 5′- AT TGCTCGTTCTTCTTATC-3′; LincRNA-p21-R, 5′-CCCTG GACCTCATTACTT-3′. H19-F, 5′-TCCAGAAAGAGGGA GTTG-3′; H19-R, 5′-GAAGCCAGACCCAGTAAG -3′. HOTAIR-F, 5′-ACCCACCAGATAAGATACAAAT-3′;* and *HOTAIR-R, 5′-CACAGCATCAATACCTCCCT-3′.* All of the primers were synthesized by Invitrogen. qRT-PCR was performed with an Applied Biosystems 7300HT machine and MaximaTM SYBR Green/ROX qPCR Master Mix (Fermentas). Fold changes in expression of each gene were calculated by a comparative threshold cycle (Ct) method using the formula 2^−(ΔΔCt)^. And the absolute expression in each subject was calculated with a standard curve, and the mean value of the control group was determined. The relative values between each sample and the mean values were calculated.

### Western blots

Total cell lysates were prepared with a detergent buffer, as described [55]. Protein concentrations were measured with the BCA Protein Assay according to the manufacturer's manual (Beyotime Institute of Biotechnology, Shanghai, China). Equal amounts (80 μg) of protein were separated by 10% sodium dodecyl sulfate-polyacrylamide gel electrophoresis and were transferred to polyvinylidene fluoride membranes (Millipore, Billerica, MA). Membranes were incubated overnight at 4°C with a 1:1000 dilution of anti-GAPDH (Sigma) and antibodies for HIF-2α (Abcam), VHL protein (Cell Signaling Technology, Beverly, MA), and ubiquitin (Novus, Littleton, CO). After additional incubation with a 1:1000 dilution of an anti-immunoglobin horseradish peroxidase-linked antibody for 1 h, the immune complexes were detected by enhanced chemiluminescence (Cell Signaling Technology). For densitometric analyses, protein bands on the blots were measured by the use of Eagle Eye II software.

### Determination of cell proliferation

Cell proliferation was evaluated by WST-8 (a tetrazolium salt that is cleaved to formazan) hydrolysis using cell counting kit-8 (Dojindo Molecular Technologies, Inc.), as described previously [56]. Briefly, cells were seeded into 96-well tissue culture plates at 4000 cells per well. Plates were incubated for 24 h at 37°C with 5% CO_2_ in a humidified incubator. After the cells were treated and incubated, 20 μL of WST-8 was added to each well, and the incubation was continued for an additional 3 h. Samples from at least three independent experiments were analyzed in duplicate. The relative cell proliferation ratios were plotted with non-treated controls to determine the 100% activity level.

### Growth kinetics

Control and arsenite-treated L-02 cells were seeded in 6-well plates at a concentration of 1 × 10^5^ per well. The plates were incubated at 37°C under 5% CO_2_ in RPMI-1640 medium supplemented with 10% FBS for 24, 48, or 72 h, then collected by trypsinization. Cells were counted in triplicate using a hemocytometer under a microscope. The population doubling time was obtained by the formula: TD = *T* × log2/(log*N*_t_ − log*N*_0_), where *N*_t_ is the inoculum cell number, *N*_0_ is the cell harvest number, and t is the time of the culture (in h) [4].

### RNA interference

Transfections of L-02 cells were performed with the N-TER™ and AccuTarget TMN nanoparticle siRNA Transfection System (Sigma, BIONEER) following the manufacturer's protocol. Briefly, 5 × 10^5^ cells were seeded into each well of 6-well plates, 18–24 h prior to transfection. The siRNA nanoparticle preparations were made by adding target gene siRNA dilutions to N-TER or AccuTarget peptide dilutions. Control siRNA and HIF-2α siRNA were purchased from Santa Cruz Biotechnology (Santa Cruz, CA). Control siRNA and MALAT1 siRNA were purchased from BIONEER.

### Cell transfection

The plasmid of HIF-2α was a gift from Dr. Rui Chen (Department of Internal Medicine, University of Texas Southwestern Medical Center at Dallas, USA). Cells were transiently transfected by use of the Lipofectamine 2000 reagent (Invitrogen, Carlsbad, CA, USA) according to the manufacturer's protocol. At 24 h after transfection, cells were treated, harvested, and used for experiments.

### ELISA assays

To determine the amounts of inflammatory cytokines in CD1 mouse serum and bronchoalveolar lavage fluid (BALF), ELISA tests were performed according to the manufacturer's instructions. A human-specific interleukin (IL)-6, IL-8, IL-1β and TNF-α ELISA from Beijing 4A Biotech Co., Ltd (Beijing) was used to determine the amounts of inflammatory cytokines. All assays were performed in duplicate and repeated three times.

### Anchorage-independent growth

Soft-agar dishes were prepared with under-layers of 0.70% agarose in RPMI-1640 or DMEM medium supplemented with 10% FBS. To test their capacity for soft-agar growth, cells were plated in triplicate at a density of 1 × 10^4^ in 2 ml of 0.35% agarose. Cultures were fed every 3 days. After 14 days, the colonies were observed under a microscope, and colonies with diameters > 80 μm were counted. These represent colonies with > 30 cells.

### Co-immunoprecipitation

Cells were extracted for 30 min with lysis buffer. After centrifugation of the preparations, the supernatants were incubated with HIF-2α antibody and subsequently with A+G Sepharose beads (Sigma) at 4°C overnight. The pellets were washed three times, re-suspended in SDS sample buffer, and boiled to remove protein from the beads. The immunoprecipitates were analyzed by Western blots with anti-ubiquitin, HIF-2α, and VHL antibodies.

### Chromatin immunoprecipitation (ChIP)

ChIP was performed as previously described [57]. Cells (1 × 10^7^) were cross-linked in 1% formaldehyde for 10 min. After cell lysis, the chromatin was fragmented to an average size of 500 bp and enriched with a magnetic Dynal bead (Invitrogen)-coupled antibody against HIF-2α or with isotype IgG at 4°C overnight. The cross-links for the enriched and the input DNA were then reversed, and the DNA was cleaned by RNase A (0.2 mg/mL) and proteinase K (2 mg/mL) before phenol/chloroform purification. The specific sequences from immunoprecipitated and input DNA were determined by PCR primers for MALAT1 promoter upstream regions: MALAT1 promoter (containing HRE1) forward, 5′- TGA GGCTGGAGTGCAGTGGC -3′, and reverse, 5′- AGGGA GGCGGAGGTTACGGT -3′, the amplicon size was 55 bp; MALAT1 promoter (containing HRE2) forward, 5′- GGAG ATTTTGTGATTTGC -3′, and reverse, 5′- TGACAGAA GTGCTGGAGA -3′, the amplicon size was 114 bp; MA LAT1 promoter (containing HRE3) forward, 5′- CCCTAA CGCCTGTGCCTGTT -3′, and reverse, 5′- CGCAGAG TAGCGACCGAGAA -3′; the amplicon size was 83 bp.

### Transwell assays

Migratory and invasive capacities of arsenite-treated L-02 cells and HCC-LM3 cells were evaluated using Transwell chambers without or with Matrigel, respectively.

### Luciferase activity assay

The pGL3-MALAT1-P1-Luc construct, the pGL3-MALAT1-P2-Luc construct, and the mut-Luc construct were purchased from Genechem (Shanghai, China). The plasmid phRL-tk (used as internal control for transfection efficiency and cytotoxicity of test chemicals) containing the Renilla luciferase gene was purchased from Promega (Madison, WI, USA). The cells proliferated to 60 – 80% confluence after 24 h of culture. Then, the cells were co-transfected with 2 μg of DNA of the reporter constructs and HIF-2α plasmid or HIF-2α siRNA using the Lipofectamine 2000 reagent (Invitrogen, Carlsbad, CA) according to the manufacturer's protocol. The amounts of luciferase and Renilla luciferase were measured with the Dual-Luciferase Reporter Assay System Kit (Promega) following the manufacturer's instructions. The values of luciferase activity for each lysate were normalized to the Renilla luciferase activity. The relative transcriptional activity was converted into fold induction above the vehicle control value.

### Lentiviral vector transfection

For knockdown of MALAT1 or HIF-2α expression, lentivirus particles carrying shRNA specifically targeting MALAT1 or HIF-2α were purchased from GeneChem (Shanghai, China). For cell infection, 60% confluent arsenite-transformed L-02 cells or HCC-LM3 cells were incubated with 50 or 30 MOI of lentivirus, respectively, and 5 μg/ml of polybrene. After 24 h of transfection, the transfection medium was replaced with medium containing puromycin (5 μg/ml; Sigma-Aldrich) for at least 2 weeks before usage to select stable cell pools. Successful transduction was determined by counting the green fluorescence emitted from GFP-stained lentiviral particles under a fluorescence microscope (EVOS FL, Advanced Microscopy Group, Mill Creek, Washington). Cells infected with lentivirus with empty vector (sh-NC) were used as controls.

### Immunohistochemistry

Immunohistochemical staining was performed on formalin-fixed, paraffin-embedded tumor samples. Sections mounted on silanized slides were de-waxed in xylene, dehydrated in ethanol, boiled in 0.01 M citrate buffer (pH 6.0) for 20 min in a microwave oven, and then incubated with 3% hydrogen peroxide for 5 min. After washing with PBS, sections were incubated in 10% normal bovine serum albumin for 5 min, followed by overnight incubation with a rabbit anti-human HIF-2α antibody and with an anti-rabbit horseradish peroxidase–conjugated secondary antibody at room temperature for 30 min. The sections were then counterstained with hematoxylin, dehydrated, cleared, and mounted.

### Statistical analyses

Derived values are presented as the means ± SD. Comparison of mean data among multiple groups was analyzed by one-way analysis of variance (ANOVA), and a multiple range least significant difference (LSD) was used for inter-group comparisons. *P* values < 0.05 were considered statistically significant. All statistical analyses were performed with SPSS 16.0.

## SUPPLEMENTARY MATERIAL FIGURES AND TABLES


