# Supplementary material for: A MALAT1/HIF-2α feedback loop contributes to arsenite carcinogenesis
Source: Oncotarget. 2015 Dec 31;7(5):5769–87. doi: 10.18632/oncotarget.6806 (PMC4868720; doi:10.18632/oncotarget.6806)
Supplement: Supplementary file 1 [file oncotarget-07-5769-s001.pdf]

A MALAT1/HIF-2α feedback loop contributes to arsenite carcinogenesis

Supplementary Materials

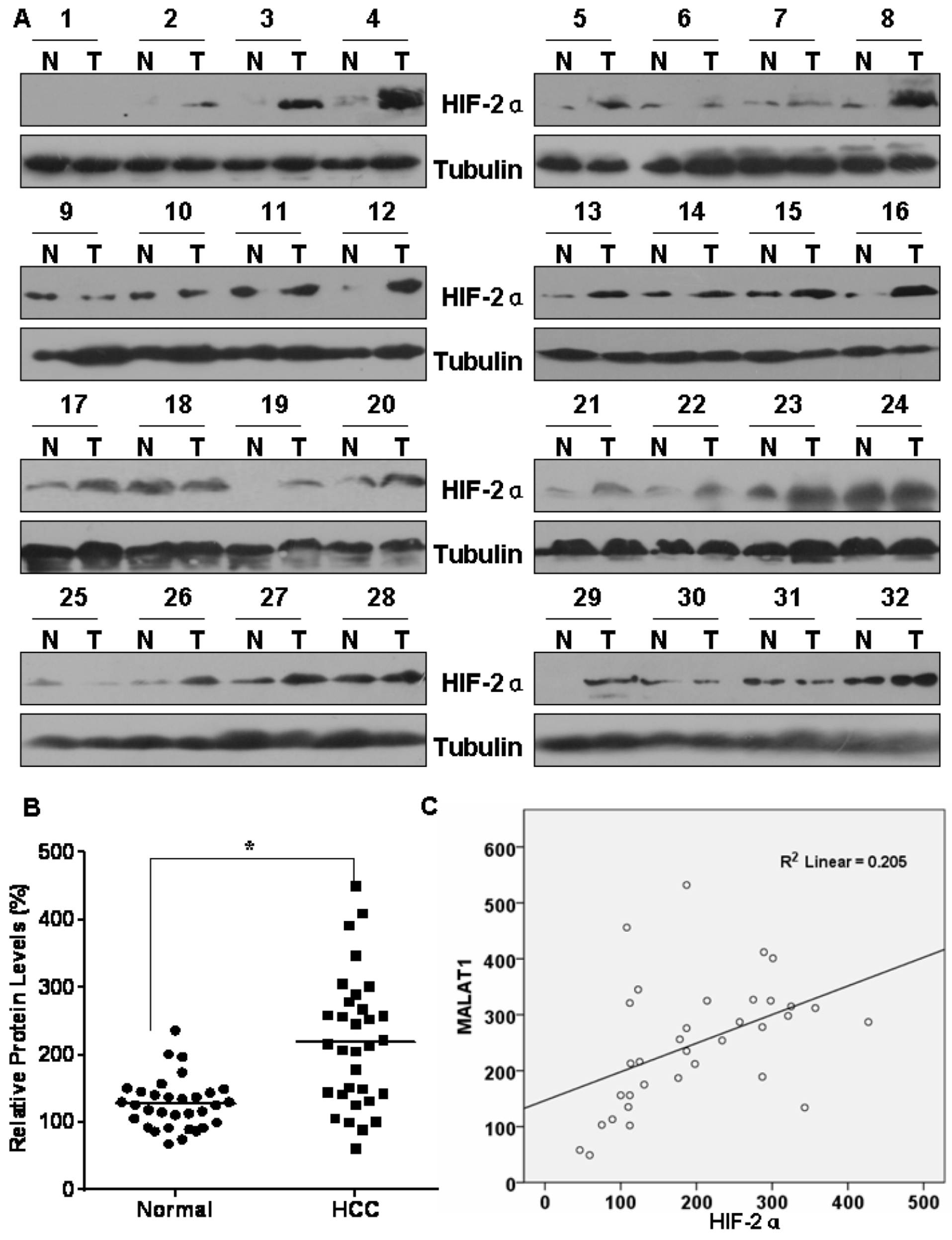

Supplementary Figure S1: (**A**) Western blots were performed and (**B**) relative protein levels (means ± SD, n = 3) of HIF-2α were determined in HCCs and in paired adjacent normal tissues. $^{*}P < 0.05$ different from adjacent normal tissues. (**C**) The relationship between MALAT1 levels and HIF-2α levels in HCC tissues (n = 32, R^2 = 0.205).

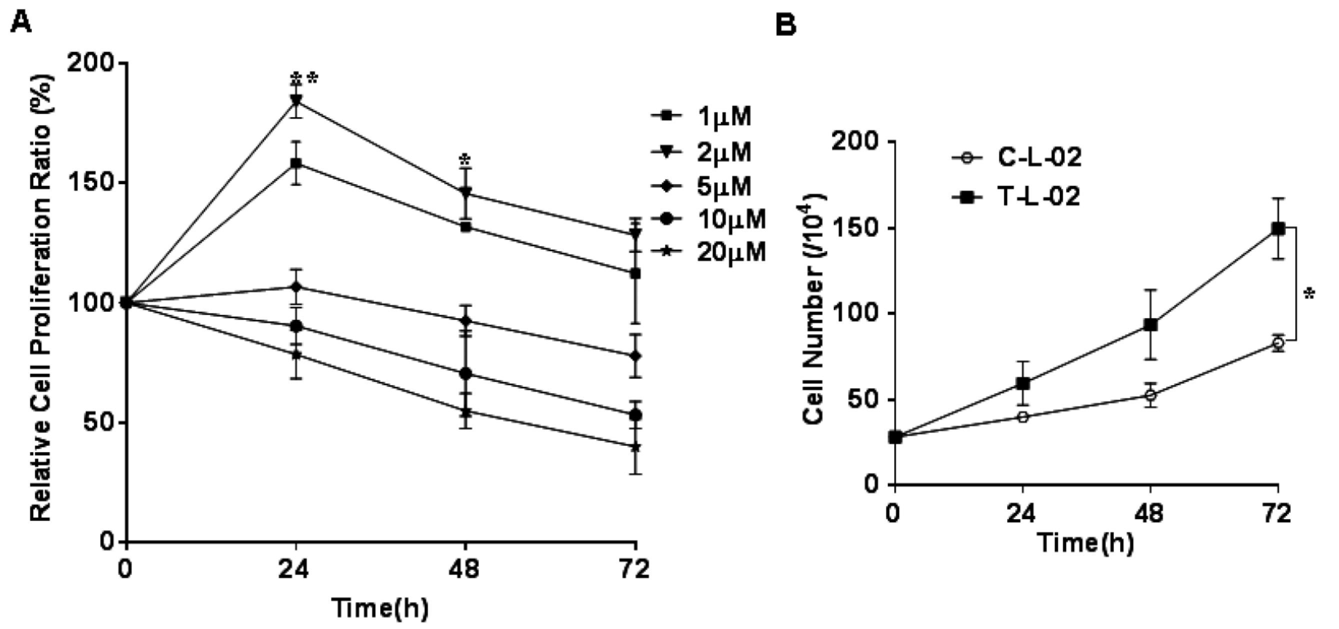

Supplementary Figure S2: L-02 cells were exposed to 0.0, 1.0, 2.0, 5.0, 10.0, or 20.0 µM arsenite for 24 h, 48 h, or 72 h. (**A**) Proliferation was measured by WST-8 hydrolysis using cell counting kit-8 assays, and the relative ratios of cell proliferation were determined by comparing to that of medium control cells (means ± SD, $n = 3$). $^{**}P < 0.01$ and $^{*}P < 0.05$ different from medium control cells. L-02 cells were exposed to 0.0 or 2.0 µM sodium arsenite for about 30 passages. (**B**) Growth curves in passage control L-02 cells and arsenite-transformed L-02 cells. There were three proliferation tests on a single batch of cells cultured for 30 passages in the presence of arsenite (means ± SD, $n = 3$). $^{*}P < 0.05$ different from medium control cells.

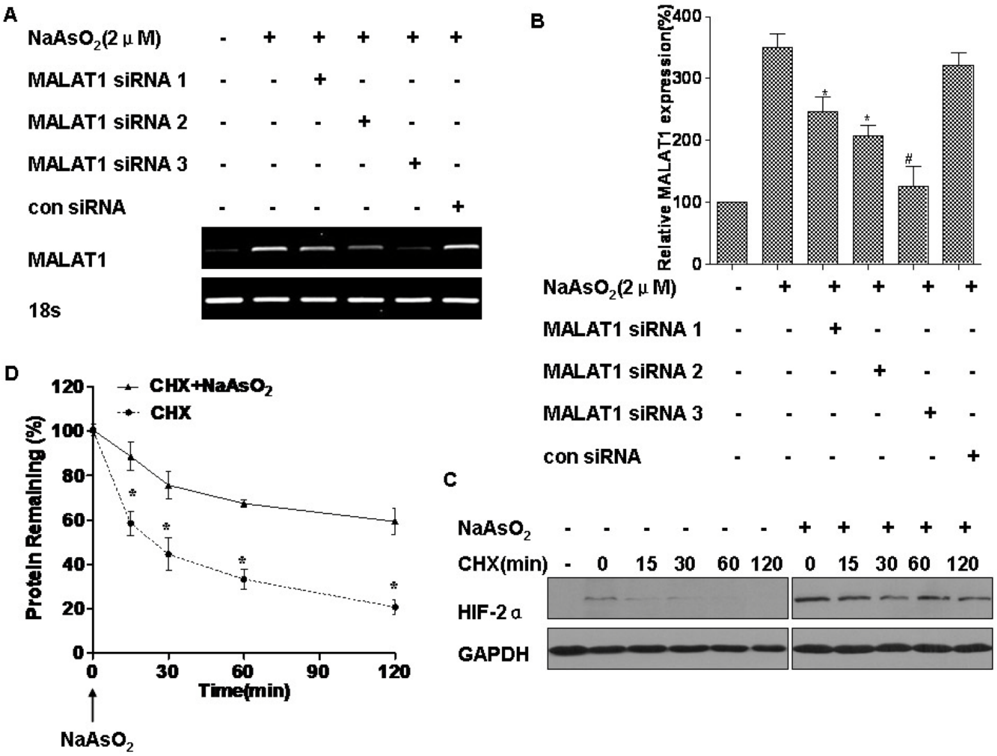

Supplementary Figure S3: L-02 cells were exposed to 100 ppm of control siRNA or MALAT1 siRNA for 24 h, then incubated with 0.0 or 2.0 µM arsenite for 24 h. (**A**) The mRNA levels of MALAT1 were measured by RT-PCR, and (**B**) the relative mRNA levels (means ± SD, $n = 3$) of MALAT1 were determined. $^{*}P < 0.05$ and $^{\#}P < 0.01$ different from arsenite-treated cells. L-02 cells were exposed to 2.0 µM arsenite for 24 h, then exposed to the protein synthesis inhibitor, CHX (10 µg/ml), in the absence or presence of arsenite for the times indicated. (**C**) Western blots were made, and (**D**) protein expression (means ± SD, $n = 3$) of HIF-2α was determined. $^{*}P < 0.05$ different from cells treated with CHX and arsenite.

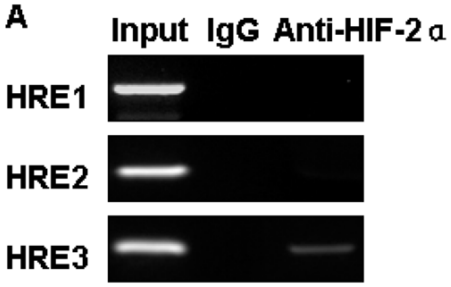

Supplementary Figure S4: (**A**) After chromatin in HCC-LM3 cells was immunoprecipitated with an antibody against HIF-2α, the binding of HIF-2α to promoters of MALAT1 at the third HRE was measured by a ChIP assay.

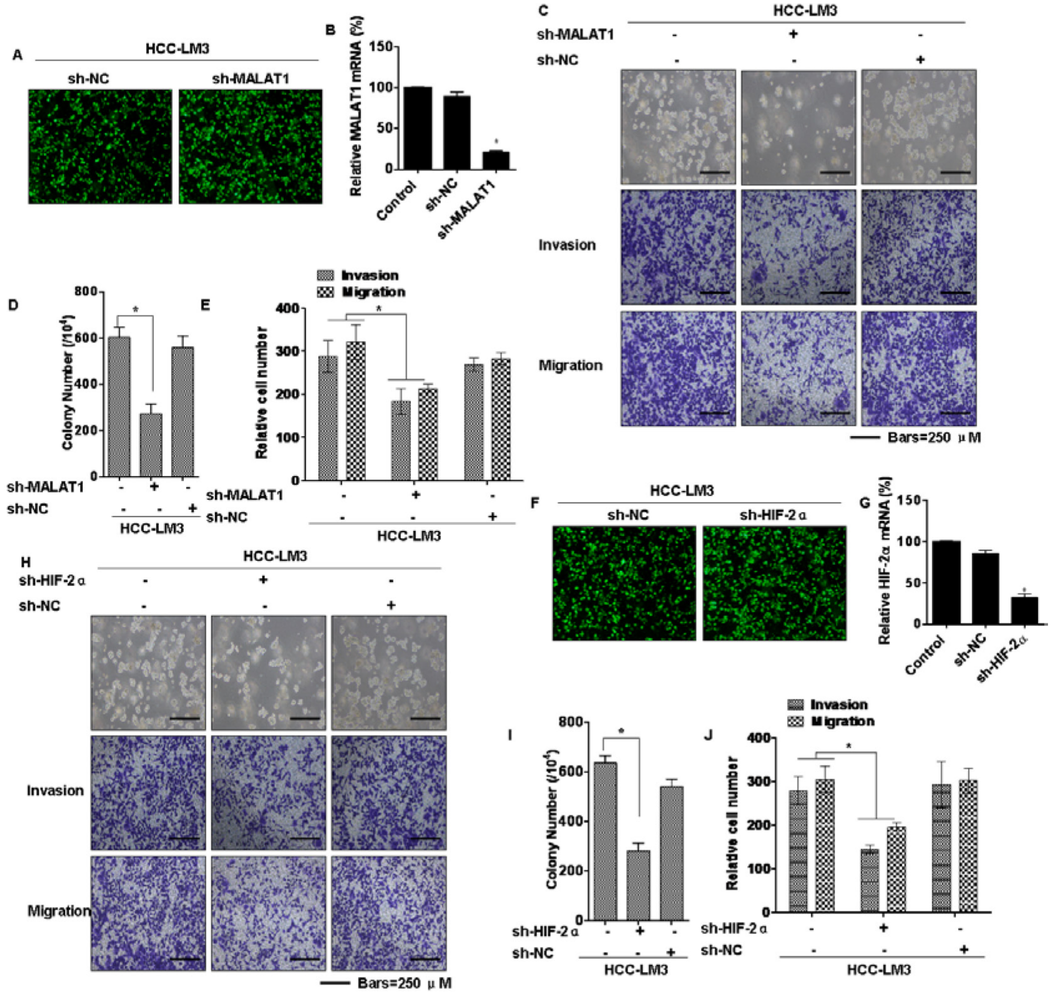

Supplementary Figure S5: HCC-LM3 cells were infected with a non-targeting control vector (sh-NC) or MALAT1 shRNA (sh-MALAT1) inducing puromycin resistance. Cells were cultured for at least 2 weeks in the presence of puromycin (5 μg/mL) before the following experiments. (**A**) HCC-LM3/shNC cells and HCC-LM3 /sh-MALAT1 cells. Fluorescent microscopy. Sh-MALAT1and sh-NC were transfected into HCC-LM3 cells. At 24 h after transfection, fluorescent microscopy showed emission green fluorescence. (**B**) qRT-PCR validated the downregulation of MALAT1 after shRNA knockdown in HCC-LM3 cells, $^{*}P < 0.05$ different from HCC-LM3 cells. (**C**) Colony formation was assessed in soft agar, and representative images of cell migration and cell invasion and (**D**) their colony numbers and (**E**) relative migrating/invading cells (means ± SD, $n = 3$) were quantified, bars = 250 μm. $^{*}P < 0.05$ different from HCC-LM3 cells. HCC-LM3 cells were infected with a non-targeting control vector (sh-NC) or HIF-2α shRNA (sh-HIF-2α), inducing puromycin resistance. Cells were cultured for at least 2 weeks in the presence of puromycin (5 μg/mL) before the following experiments. (**F**) HCC-LM3/shNC cells and HCC-LM3/sh-HIF-2α cells. Fluorescent microscopy. Sh-HIF-2α and sh-NC were transfected into HCC-LM3 cells. At 24 h after transfection, fluorescent microscopy showed emission green fluorescence. (**G**) qRT-PCR validated the downregulation of HIF-2α after shRNA knockdown in HCC-LM3 cells, $^{*}P < 0.05$ different from control HCC-LM3 cells. (**H**) Colony formation was assessed in soft agar, and representative images of cell migration and cell invasion and (**I**) their colony numbers and (**J**) relative migrating/invading cells (means ± SD, $n = 3$) were quantified, bars = 250 μm. $^{*}P < 0.05$ different from HCC-LM3 cells.

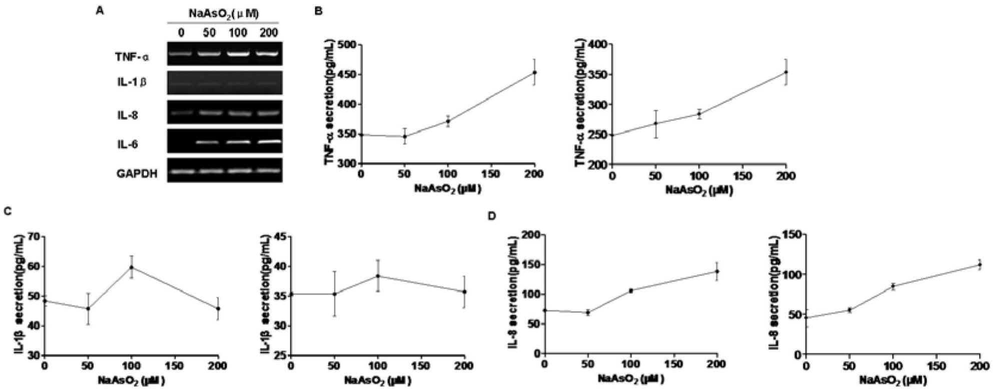

Supplementary Figure S6: Densities of bands were quantified by Eagle Eye II software. 18s ribosomal RNA levels, measured in parallel, served as controls. The four groups of CD1 mice were untreated or treated with low, middle, or high concentrations (50 μM, 100 μM, or 200 μM, respectively) of arsenite, which were added to the drinking water daily for 3 months. (**A**) The mRNA levels of TNF-α, IL-1β, IL-6, and IL-8 were determined by RT-PCR for the hepatic tissues of mice treated with low, middle, or high concentrations (50 μM, 100 μM, or 200 μM, respectively) of arsenite. (**B**, **C**, and **D**) The levels of TNF-α, IL-1β, and IL-8 present in the BALF and serum (means ± SD, n = 3) were measured by ELISA.

Supplementary Table S1: RT-PCR Primer sequences used

18s	*5′-GTAACCCGTTGAACCCCATT-3’*
	5′-CCATCCAATCGGTAGTAGCG-3’
MALAT1	*5′-TCCAGAAAGAGGGAGTTG-3’*
	5′-GAAGCCAGACCCAGTAAG-3’
GAS5	*5′-ACTGCCTCACAACGTTTGTGCCT-3’*
	5′-CCAGCGTGAGCTCCAGTGCTTT-3’
HOTAIR	*5′-ACCCACCAGATAAGATACAAAT-3’*
	5′-CACAGCATCAATACCTCCCA-3’
LincRNA-p21	*5′-ATTGCTCGTTCTTCTTATC-3’*
	5′-CCCTGGACCTCATTACTT-3’
HIF-2α	*5′-CACCAAGGGTCAGGTAGTAAG-3′*
	5′-GGTTGCGAGGGTTGTAGAT-3′
VEGF	*5′- AGAAGGAGGAGGGCAGAATC -3′*
	5′- ACACAGGATGGCTTGAAGATG -3′
Oct4	*5′- GCTTCCTCCACCCACTTCT -3′*
	5′- GTATTCAGCCAAACGACCAT-3′
IL-6	*5′-AGTAGTGAGGAACAAGCCAGA-3′*
	5′-TACATTTGCCGAAGAGCC-3′
IL-8	*5′-ACTTCTCCACAACCCTCTG-3′*
	5′-ACTCCAAACCTTTCCACC-3′
IL-1β	*5′-ACAGTGGCAATGAGGATG-3′*
	5′-TGTAGTGGTGGTCGGAGA-3′
TNF-α	*5′-TTGAAGAGGACCTGGGAGTAGAT-3′*
	5′-CGAGTGACAAGCCTGTAGCC-3′
GAPDH	*5′-GCATCCTGGGCTACACTG-3’*
	5′-TGGTCGTTGAGGGCAAT-3’

Supplementary Table S2: Survival and body weights of CD1 mice exposed to arsenite for 3 months

Dose (µM)	Initial group size	Survival at 3 months	Body weight (g)	
			7 weeks	3 months
0	16	16	34.5 ± 3.25	46.9 ± 3.72
50	16	16	32.9 ± 2.59	49.0 ± 4.53
100	16	16	33.6 ± 1.98	47.1 ± 4.75
200	16	16	34.4 ± 1.64	46.8 ± 4.66
